# Statins for primary prevention of cardiovascular events in people with HIV: target trial and modelling study

**DOI:** 10.1136/bmjmed-2024-001132

**Published:** 2025-05-08

**Authors:** Henock G Yebyo, Huldrych F Günthard, Eva A Rehfuess, Nicola Serra, Sarah R Haile, Oliver Senn, Gregory M Lucas, Oliver Langselius, Jennifer E Thorne, Vincent C Marconi, Sally B Coburn, Raynell Lang, Jonathan A Colasanti, Michael J Silverberg, Sonia Napravnik, Mona Loutfy, Maile Karris, Timothy R Sterling, Greer A Burkholder, Keri N Althoff, Milo A Puhan

**Affiliations:** 1 Epidemiology, Biostatistics, and Prevention Institute, University of Zurich, Zurich, Switzerland; 2 Department of Infectious Diseases and Hospital Epidemiology, University Hospital Zurich, Zurich, Switzerland; 3 Institute of Medical Virology, University of Zurich, Zurich, Switzerland; 4 Institute for Medical Information Processing, Biometry, and Epidemiology, Ludwig-Maximilians-Universität München, Munich, Germany; 5 Pettenkofer School of Public Health, Ludwig-Maximilians-Universität München, Munich, Germany; 6 Physics Institute, University of Zurich, Zurich, Switzerland; 7 European Organisation for Nuclear Research, Geneva, Switzerland; 8 Institute of Primary Care, University of Zurich and University Hospital Zurich, Zurich, Switzerland; 9 Division of Infectious Diseases, Department of Medicine, School of Medicine, Johns Hopkins University, Baltimore, Maryland, USA; 10 Bloomberg School of Public Health, Johns Hopkins University, Baltimore, Maryland, USA; 11 School of Medicine and Rollins School of Public Health, Emory University, Atlanta, Georgia, USA; 12 Department of Epidemiology, Bloomberg School of Public Health, Johns Hopkins University, Baltimore, Maryland, USA; 13 Department of Medicine, University of Calgary, Calgary, Alberta, Canada; 14 Department of Medicine, Grady Health System, Emory University, Atlanta, Georgia, USA; 15 Division of Research, Kaiser Permanente, Oakland, California, USA; 16 Division of Infectious Diseases, University of North Carolina at Chapel Hill, Chapel Hill, North Carolina, USA; 17 Maple Leaf Medical Clinic, Women’s College Hospital, Toronto, Ontario, Canada; 18 Department of Medicine, University of California San Diego, La Jolla, California, USA; 19 Vanderbilt University Medical Center, Nashville, Tennessee, USA; 20 Division of Infectious Diseases, University of Alabama at Birmingham, Birmingham, Alabama, USA

**Keywords:** Preventive medicine, Cardiology, Hiv

## Abstract

**Objective:**

To evaluate the effectiveness and benefit-harm balance of various statins for the primary prevention of cardiovascular disease in people with HIV.

**Design:**

Target trial and modelling study.

**Setting:**

North American AIDS Cohort Collaboration on Research and Design (NA-ACCORD), 1995 to 2019. NA-ACCORD integrates individual level data from >20 HIV cohorts across the US and Canada from people with HIV who have successfully linked into care.

**Participants:**

157 699 people with HIV enrolled in one of the cohorts of NA-ACCORD. 54 165 eligible individuals, aged 40-75 years, were enrolled in the target trial.

**Main outcome measures:**

The primary outcomes for the target trial were the 10 year effects of statins on cardiovascular disease events (fatal and non-fatal myocardial infarction, hospital admission for unstable angina, coronary or arterial revascularisation, fatal and non-fatal stroke, or transient ischaemic attack) and harm outcomes (type 2 diabetes, mild cognitive impairment, rhabdomyolysis, and myopathy). The secondary outcome was the 10 year risk threshold where the reduction in cardiovascular disease outweighed the increased risk of harm outcomes, showing an overall net benefit of statins.

**Results:**

Participants who first started receiving treatment with statins (statin initiators) had a 21% reduction in cardiovascular disease events (hazard ratio 0.79, 95% confidence interval (CI) 0.72 to 0.87) and a 26% reduction in the combined risk of stroke and myocardial infarction (0.74, 0.56 to 0.98), but a 12% increase in the risk of type 2 diabetes (1.12, 1.01 to 1.25) compared with participants who developed the indication but did not take statins (non-initiators). The effects on cognitive impairment (hazard ratio 1.13, 95% CI 0.82 to 1.56), myopathy (1.10, 0.76 to 1.61), and rhabdomyolysis (1.09, 0.68 to 1.75) were not statistically significant. On average, the benefit of statins exceeded harms for individuals with a 10 year baseline risk of cardiovascular disease of ≥13.8%. Subgroup specific thresholds included men (14.2%), women (11.1%), ages 40-64 years (13.8%) versus 65-75 years (15.1%), and CD4 count >200 cells/mm³ (13.6%) versus <200 cells/mm³ (15.3%). Varying weights for cardiovascular disease yielded thresholds ranging from 11.6% to 54.0%, whereas weights for harm outcomes resulted in thresholds ranging from 5.0% to >30.0%.

**Conclusions:**

In this study, statins benefitted individuals with HIV with a moderate or high risk of cardiovascular disease, but the threshold for net benefit varied by patient subgroup and preference, implying the need to customise statin treatment to individual risks, preferences, and treatment goals. Given the limitations of observational data, further controlled studies are needed to evaluate the efficacy and safety of statins in people with HIV receiving modern antiretroviral therapy.

**WHAT IS ALREADY KNOWN ON THIS TOPIC**

- REPRIEVE (Randomised Trial to Prevent Vascular Events in HIV) investigated the effect of pitavastatin on cardiovascular disease and safety outcomes
- Robust evidence is lacking on the effectiveness and safety of different statins for preventing cardiovascular disease in people with HIV

**WHAT THIS STUDY ADDS**

- Estimates of the effectiveness and safety of the use of statins for the primary prevention of cardiovascular disease are provided, based on a large observational cohort of adults receiving HIV care in the US and Canada
- Participants who initiated statin treatment had lower cardiovascular disease event rates but higher diabetes rates than participants who developed the indication but did not take statins
- Cardiovascular disease risk thresholds were identified by statin type, subgroup, and patient preference, emphasising the importance of customising statin treatment to optimise benefits

**HOW THIS STUDY MIGHT AFFECT RESEARCH, PRACTICE, OR POLICY**

- The findings suggest that statins benefit people with HIV but highlight the need for careful evaluation of the net benefits based on baseline risk of cardiovascular disease, potential harms, and patient preferences, rather than a one-size-fits-all approach
- Controlled studies on various statins in people with HIV taking modern antiretroviral therapy regimens are essential, because earlier observational data might have limitations that could affect the observed benefits

## Introduction

 The risk of cardiovascular disease in people with HIV is twice as high as in those without HIV.^1^ As well as the traditional risk enhancers, chronic immune activation and inflammation related to HIV and some antiretroviral therapy, particularly protease inhibitors and early generations of nucleoside reverse transcriptase inhibitor analogues, contribute to progressive atherosclerosis and unfavourable lipid dysregulation in people with HIV.^2^ Newer antiretroviral treatments, such as the integrase strand transfer inhibitors and tenofovir alafenamide fumarate, are associated with weight gain and subsequent metabolic changes that might further increase the risk of cardiovascular disease.^3^,^5^

Preventive strategies for cardiovascular disease in people with HIV are limited by insufficient evidence.^6^ Treatment with statins is a mainstay of lipid lowering and cardiovascular risk prevention in the general population.^7 8^ Although guidelines recommend single thresholds for starting statins, focused mainly on cardioprotective effects, our previous study found that these thresholds varied between 14% and 22%, depending on age and sex, with a benefit-harm trade-off analysis.^9^ Statins, however, have not been well evaluated in people with HIV. REPRIEVE (Randomised Trial to Prevent Vascular Events in HIV)^10^ was the first trial that tested a daily dose of 4 mg of pitavastatin.^2^ The trial showed a 35% reduction in cardiovascular disease but increased risks for diabetes and muscle disorders, indicating the need for a careful evaluation of the benefit-harm balance. The effects of other commonly used statins should be examined because of potential differences in effectiveness and safety. Statins should provide benefits for people with HIV by reducing lipid levels^10 11^ and also by pleiotropic effects, such as reducing inflammation and markers that enhance the risk of atherosclerosis.^12^ The harms, however, including interactions with antiretroviral therapy, hepatitis C virus protease inhibitors, and CYP3A4 (cytochrome P450 3A4) inhibiting antimicrobial agents, could offset the cardioprotective effects in certain individuals.^2 13^ Hence as well as evaluating effectiveness and harms, a careful and systematic approach for personalised decisions is needed to identify individuals who would derive most benefit from statins based on their risk for the treatment-related benefit and harm outcomes and antiretroviral therapy.

In this study, we used data from the North American AIDS Cohort Collaboration on Research and Design (NA-ACCORD),^14^ the largest collaboration of longitudinal HIV cohorts, to estimate the effectiveness and harms of statins for the primary prevention of cardiovascular disease, with a target trial approach.^15^ We also evaluated the balance between cardiovascular disease events prevented and the harms of statins, and determined cardiovascular disease risk thresholds above which the benefits outweighed the harms. The approach could inform how decision making should be guided to optimise the benefits of statins by identifying individuals or subgroups who would benefit most based on their risks and preferences.

## Methods

### Study design

We developed an open label target trial protocol and emulated it with observational data from NA-ACCORD to evaluate the effects of statins on the incidence of primary cardiovascular disease and harm outcomes in people with HIV. Table 1 gives specifications of the target trial.

**Table 1:** Target trial specifications and emulation to estimate effectiveness and safety of statins for primary prevention of cardiovascular disease

Protocol component	Target trial	Target trial emulation
Design	Open label trial	Same as for target trial. Because individuals know what treatments they are taking in real world practice, the target trial we emulated was open label
Eligibility criteria	Age 40-75 years	Same as for target trial
	No previous history of cardiovascular disease, no statin treatment started before enrolment LDL <4.9 mmol/L, total cholesterol <7.5 mmol/L, and 10 year cardiovascular disease risk score <7.5 for individuals with LDL 1.8-4.9 mmol/L and triglycerides <5.7 mmol/L, or LDL <1.8 mmol/L for patients with diabetes No contraindications at baseline, including cognitive impairment, HIV dementia, renal and liver disease, muscle disorders, history of allergy or severe adverse reactions to statins, liver enzymes >3 times the upper value of a normal range, cancer 12 months before study entry, and no current use of erythromycin, clarithromycin, colchicine, or fluconazole	Same as for target trial
Treatment strategies and start of follow-up	Statin treatment initiation *v* non-initiation	Statin initiation was the first date of a statin prescriptionNon-initiation (controls) had indications for statin treatment: LDL ≥4.9 mmol/L, total cholesterol ≥7.5 mmol/L, and 10 year cardiovascular disease risk score ≥7.5 for individuals with LDL 1.8-4.9 mmol/L and triglycerides ≥5.7 mmol/L, or LDL ≥1.8 mmol/L for patients with diabetesFollow-up began at these time points: when statin treatment was first started (initiators) or time participants developed the indication but did not take statins (non-initiators)
Randomisation	Individuals randomly assigned to a strategy at baseline and are aware of their assigned strategy	Emulated randomisation by adjusting for baseline confounders determined when treatment assigned (statin initiation or developed indications for statin treatment), including age, sex, race, cohort, year of enrolment to NA-ACCORD, 10 year risk score, hypertension, diabetes, LDL/total cholesterol, systolic blood pressure, family history of cardiovascular disease, behavioural risk, cohort site, smoking, year of enrolment, CD4 count, viral load, and use of abacavir, protease inhibitors, nucleoside reverse transcriptase inhibitor analogues, integrase strand transfer inhibitors, tenofovir alafenamide fumarate, and antihypertensive treatments
Ending follow-up	Follow-up ended on the date of occurrence of the first event of interest (cardiovascular disease, diabetes, or cognitive impairment), death, loss to follow-up, or censoring at 10 years after enrolment in the target trial, whichever occurred first	Individuals exited the study at the first occurrence of: outcomes of interest; date of death; loss to follow-up (defined as a period of ≥2 years without CD4 or viral load measurement); 10 year follow-up after entry to the target trial; closing of cohorts or comorbidity observation window; or administrative censoring (31 December 2019)
Outcomes	Time to any cardiovascular disease events (fatal and non-fatal myocardial infarction, fatal and non-fatal stroke, hospital admission for unstable angina, or coronary or peripheral arterial revascularisation) and harm outcomes (diabetes, mild cognitive impairment, and rhabdomyolysis)	Same as for target trialOutcome assessment was not blinded, and not all outcomes were determined in all NA-ACCORD cohorts
Causal contrast	Intention-to-treat effect	Observational analogue intention-to-treat average treatment effect
Statistical analysis	Intention-to-treat analysis	Same as for target trial

LDL, low density lipoprotein; NA-ACCORD, North American AIDS Cohort Collaboration on Research and Design.

NA-ACCORD integrates individual level data from >20 HIV cohorts across the US and Canada from people with HIV who have successfully linked into care (defined as ≥2 HIV clinical visits in 12 months).^14^ Our study included 157 699 people with HIV enrolled in one of these cohorts between 1995 and 2019 (online supplemental material). NA-ACCORD uses rigorous data collection and harmonisation methods, and quality checks, and follows standardised data management processes to ensure data reliability. Although the cohorts represent wide geographic areas across both countries,^14^ differences in population personal characteristics, healthcare access, and care delivery should be considered when interpreting findings and applying them to other settings.

### Participants and eligibility

We defined the entry date for follow-up based on the start of combination antiretroviral therapy (1 January 1995), enrolment in NA-ACCORD, the opening date of the specific cohort, or the start of the observation windows for comorbidities (such as diabetes, hepatitis C virus, and body mass index), whichever occurred later. We then included individuals aged 40-75 years with no previous cardiovascular disease events, and no previous use of statins or indications for statin use, specifically those with low density lipoprotein levels <4.9 mmol/L, total cholesterol <7.5 mmol/L, triglycerides <5.7 mmol/L, and 10 year predicted cardiovascular disease risk score <7.5% based on the pooled cohort equations for those with low density lipoprotein levels of 1.8-4.9 mmol/L, or low density lipoprotein levels <1.8 mmol/L for participants with diabetes at entry to follow-up.^16 17^

Exclusion criteria were CD4 count <100 cells/mm^3^, use of other lipid lowering agents (such as ezetimibe and proprotein convertase subtilisin/kexin type 9 (PCSK-9) inhibitors) within 60 days of study entry, alanine aminotransferase levels >3 times the upper limit of normal, renal diseases, myopathy, rhabdomyolysis, or dementia within 60 days of study entry, cancer within 12 months of entry to the target trial, or current use of treatments with potential interactions with statins. Participants were assessed at the time of entry to follow-up.

We defined the start of statin treatment as the first statin prescription (statin initiators). Controls (non-initiators) were individuals who first developed an indication for statin therapy: low density lipoprotein levels ≥4.9 mmol/L, total cholesterol levels ≥7.5 mmol/L, and a 10 year cardiovascular disease risk of ≥7.5% for those with low density lipoprotein levels 1.8-4.9 mmol/L, or in patients with diabetes, low density lipoprotein levels ≥1.8 mmol/L, and having no contraindications. We included all statin types, but excluded simvastatin and lovastatin because of their higher risk of interactions.^13 18^ Individuals exited the study at the earliest occurrence of any of the following: outcomes of interest, death, loss to follow-up (defined as a period of ≥2 years without a CD4 or viral load measurement), reaching a 10 year follow-up after entry to the target trial, closing of cohorts or observation window for comorbidities, or administrative censoring at 31 December 2019.

### Outcomes

The primary outcome was the first cardiovascular disease event (fatal and non-fatal myocardial infarction, hospital admission for unstable angina, coronary or arterial revascularisation, fatal and non-fatal stroke, or transient ischaemic attack). Statin harm outcomes included ICD (international classification of diseases) diagnosis codes for type 2 diabetes, mild cognitive impairment (non-HIV dementia), rhabdomyolysis, and myopathy (defined as creatine kinase levels >10 times the upper limit of normal) (online supplemental table S1).^19^

### Statistical analysis

We evaluated the effectiveness and safety of statins. Participants who first started receiving treatment with statins (statin initiators) were closely matched with participants who developed the indication but did not take statins (non-initiators). Matching factors were personal characteristics (age, sex at birth, race and ethnic group, and HIV acquisition risk), risk factors for the outcomes of interest (body mass index, smoking, cholesterol levels, 10 year cardiovascular disease risk score, family history of cardiovascular disease, hypertension, use of antihypertensive drug treatments, systolic blood pressure, diabetes, and hepatitis C virus infection), and factors related to HIV (CD4 count, viral load, and use of abacavir, protease inhibitors, integrase strand transfer inhibitors, and tenofovir alafenamide fumarate). The cohort indicator was also considered in matching, to account for variations in practice, as well as year of enrolment, to account for changes in specifications for antiretroviral therapy and guidelines over time. We imputed missing covariates with multiple imputations before matching. We also accounted for the competing risk of death with inverse probability weighting.

Statin initiators were matched with one to three of their closest non-initiators, based on their propensity to receive statins, taking into account the wide range of covariates listed above, as well as the competing risk of death. We evaluated the covariate balance with a plot of the standardised mean differences between variable values for statin initiators and non-initiators, with a difference of ≤0.1 considered to be acceptable matching (online supplemental figures S1–S5).

We fitted a Cox proportional hazards model to the matched cohorts, with the use of statins as the intervention, incorporating matching weights for multiple controls. We estimated the 10 year cumulative incidence for each outcome with the Kaplan-Meier estimator for statin initiators and non-initiators. The observational analogue, intention-to-treat hazard ratios of starting statins, on cardiovascular disease, diabetes, cognitive impairment, myopathy, and rhabdomyolysis were estimated with Cox proportional hazard models.

### Sensitivity analysis

We performed sensitivity analyses with different alternative assumptions to assess the robustness of the results. Firstly, because a statin adherence indicator was not available, we restricted the analysis to individuals who were prescribed statins for >12 months versus never users, which might serve as a proxy indicator for adherence. Secondly, a random placebo treatment was assigned, disregarding patient characteristics, to assess the expected null effect in the absence of residual confounders. Thirdly, analyses were performed separately for specific statin types, including atorvastatin, rosuvastatin pravastatin, and fluvastatin. Finally, we estimated subgroup effects based on factors defined a priori, including age, cardiovascular disease risk score, hypertension, use of integrase strand transfer inhibitors, and diabetes, among others.

For the benefit-harm analysis, we weighed prevented cardiovascular disease events against cumulative harm events, including diabetes, myopathy, rhabdomyolysis, and cognitive impairment, as estimated in our target trial. We included all potential outcomes in the benefit-harm analysis, irrespective of whether they were significant, and accounted for the statistical uncertainty of the estimates. Also, we incorporated statin related outcomes from the general population, that were not estimable from the NA-ACCORD data, specifically hepatic dysfunction, renal dysfunction, and cataracts from external sources.^720^,^22^ We assessed the relative effects of only these three outcomes from external sources, while their outcome risks were taken from NA-ACCORD.

The benefit-harm analysis considered the relative treatment effect, outcome risks in participants who were not treated with statins, and preferences. The cumulative risk differences with and without statins were predicted with an exponential model adjusted for the competing risk of death. We weighed the risk difference by their respective preference (relative importance) values to summarise the benefits and harms in one number. Preference is also an important factor to consider for treatment decisions. It can be a proxy indicator for the patient's willingness to accept or reject (ie, risk aversion) the risks of harm in pursuit of the benefit. Because we did not find studies on outcome preferences specific to people with HIV, we used preferences from a previous study conducted in Ethiopia and Switzerland in people without HIV.^23^ We adjusted these values to the outcome features in our study by applying different anchor values. Preference weights ranged from 0.0 for outcomes with no perceived concern to 1.0 for the worst outcome (ie, death for most people). We conducted extensive analyses with a range of preferences to examine the preference sensitivity of the results.

We summed the preference adjusted risk differences across all outcomes to derive the benefit-harm index distribution, with negative (harm outweighed benefit), positive (benefit outweighed harm), or zero (equipoise) values. We repeated this process 100 000 times, accounting for the statistical uncertainty around the input parameters. Since the aggregated index might not be directly interpretable, we provided a proxy interpretation by transforming the index to cardiovascular disease equivalent events (online supplemental material). The analyses included bootstrapping with 1000 replicate samples to estimate 95% uncertainty intervals based on the 2.5th and 97.5th percentiles in the distributions of net benefit and outcome events.

For a more intuitive interpretation, we calculated the probability of the benefits of statins outweighing the harms (ie, net benefit) from the distribution of the benefit-harm balance index. We conducted this analysis for 10 year baseline risk values of cardiovascular disease ranging from 0% to 30% and identified the threshold at which the probability of net benefit reached at least 0.6. We selected a probability of 0.6 for defining the risk threshold to ensure a non-zero minimal net benefit, rather than using a probability of 0.5 where the expected net benefit would be zero. We also provided the thresholds defined at 0.5 probability of net benefit. The method is described in our previous works,^9 24 25^ and the online supplemental material gives more details.

We conducted the analyses for varying hypothetical weights, different time horizons, and subgroups based on sex, age, body mass index, race, use of protease inhibitors or integrase strand transfer inhibitors, hepatitis C virus infection, hypertension, viral load or CD4 count, and smoking status at entry to the target trial. Analyses were done with R software (version 4.0.4) and in accordance with standards of reporting quantitative benefit-risk models and estimates from observational studies.^26 27^

### Patient and public involvement

This study used data from NA-ACCORD. Patients or the public were not involved in the development of the research question or outcome measures, or in the design, implementation, or dissemination plans of this study because we did not have access to individual participants in the NA-ACCORD cohorts. All data were anonymised and provided under agreements that preclude patient contact. Findings will be shared through open access publication and distributed to the NA-ACCORD network.

## Results

Of the initial sample of 157 699 people with HIV, 54 165 eligible individuals were enrolled in the target trial (online supplemental figure S6). Table 2 shows the baseline characteristics of the matched sample at entry to the target trial (online supplemental table S2 describes the characteristics of individuals before matching). Nearly 99% of participants were enrolled in NA-ACCORD cohorts after 2000, with 72.0% enrolled in 2006 or later. Most participants were men (85.0%). Mean age was 50.9 years (standard deviation (SD) 8.0) for statin initiators and 50.7 years (SD 7.0) for non-initiators. Median 10 year cardiovascular disease risk scores for statin initiators and non-initiators were 8.8 (interquartile range (IQR) 5.5-11.1) and 8.6 (5.3-10.0), and low density lipoprotein levels were 3.2 mmol/L (SD 1.1) and 3.2 mmol/L (SD 0.9), respectively.

**Table 2:** Selected baseline characteristics of matched individuals at time of first use of statins for treated group (statin initiators) or at first indication for statin use for participants who did not take statins (non-initiators)

Characteristics	Statin initiators (n=8272)	Statin non-initiators (n=14 332)
Sex at birth:		
Women	1241 (15.0)	2164 (15.1)
Men	7031 (85.0)	12 168 (84.9)
Race:		
Asian	91 (1.1)	172 (1.2)
Black	3069 (37.0)	5289 (36.9)
White	3946 (47.7)	6822 (47.6)
Other	1175 (14.2)	2049 (14.3)
Ever smoker	5592 (67.6)	9674 (67.5)
Hispanic	1042 (12.6)	1849 (12.9)
Year of enrolment to target trial:		
1995-99	124 (1.4)	215 (1.5)
2000-05	2233 (26.8)	3715 (25.9)
2006-12	3559 (43.0)	6164 (43.0)
2013-19	2416 (29.0)	4238 (29.6)
Mean (SD) age (years)	50.9 (8.0)	50.7 (7.0)
Mean (SD) body mass index	27.2 (5.0)	27.2 (6.0)
Median (IQR) 10 year cardiovascular disease risk score	8.8 (5.5-11.1)	8.6 (5.3-10.0)
Mean (SD) low density lipoprotein (mmol/L)	3.2 (1.1)	3.2 (0.9)
Mean (SD) high density lipoprotein (mmol/L)	1.1 (0.4)	1.1 (0.4)
Mean (SD) systolic blood pressure (mm Hg)	125.2 (9.0)	124.7 (14.0)
CD4 (cells/mm^3^):		
≥200	7461 (90.2)	12 927 (90.2)
<200	811 (9.8)	1405 (9.8)
Hypertension	3036 (36.7)	5088 (35.5)
Use of hypertensive drug treatments	1754 (21.2)	2995 (20.9)
Type 2 diabetes	1026 (12.4)	1620 (11.3)
Family history of cardiovascular disease	99 (1.2)	172 (1.2)
Abacavir use	728 (8.8)	1290 (9.0)
Integrase strand transfer inhibitor use	819 (9.9)	1476 (10.3)
Protease inhibitor use	736 (8.9)	1333 (9.3)
Hepatitis C virus infection	149 (1.8)	287 (2.0)
HIV acquisition risk:		
Injection drug use	2283 (27.6)	4085 (28.5)
Men to men homosexual	3508 (42.4)	6005 (41.9)
Heterosexual	1167 (14.1)	1907 (13.3)
Unknown or other (haemophilia, blood transfusion, or perinatal)	1315 (15.9)	2336 (16.3)

Data are number (%) unless indicated otherwise.

The pooled cohort equations were used to estimate 10 year risk. The 10 year cardiovascular disease risk score might be overestimated because we considered ever smoker because current smoker status was not in the data.

IQR, interquartile range; SD, standard deviation.

**Figure 1:**
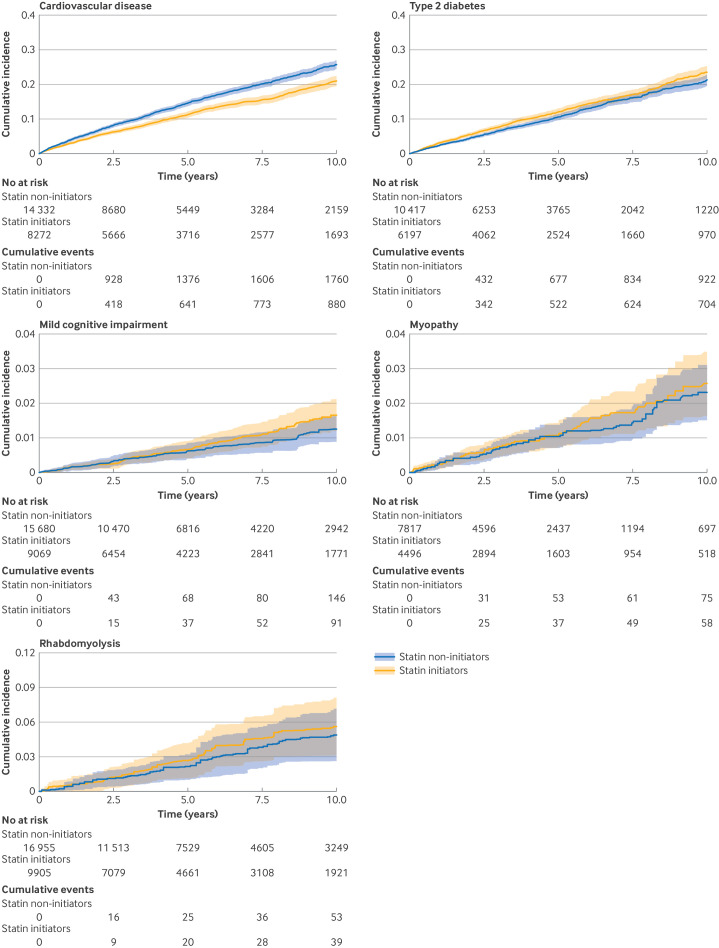
10 year cumulative incidence of cardiovascular disease events (fatal and non-fatal myocardial infarction, hospital admission for unstable angina, coronary or arterial revascularisation, fatal and non-fatal stroke, or transient ischaemic attack) and harm outcome events (type 2 diabetes, mild cognitive impairment, myopathy, and rhabdomyolysis) in participants who first started receiving statin treatment (statin initiators) and in those who developed the indication but did not take statins (non-initiators)

The 10 year cumulative risk was lower for cardiovascular disease but higher for diabetes in statin initiators than in non-initiators (table 3, figure 1, and online supplemental figure S7). Statin initiators had a 21% reduction in cardiovascular disease incident events (hazard ratio 0.79, 95% CI 0.72 to 0.87). The effect was particularly pronounced because the baseline risk of cardiovascular disease increased. For individuals with a 10 year cardiovascular disease risk of <7.5%, ≥7.5-10.0%, >10.0-15.0%, and ≥15.0%, statins had effects on cardiovascular disease risk reduction, with hazard ratios of 0.84 (95% CI 0.70 to 1.00), 0.75 (0.56 to 0.95), 0.56 (0.47 to 0.68), and 0.65 (0.55 to 0.77), respectively. The effect increased slightly with age, with a hazard ratio of 0.76 (95% CI 0.61 to 0.95) for individuals aged 60-75 years compared with 0.79 (0.71 to 0.88) for those aged 40-60 years (online supplemental table S3). The effect was greater on the combined events of myocardial infarction and stroke (hazard ratio 0.74, 0.56 to 0.98).

**Table 3:** Treatment effect on cardiovascular disease events (fatal and non-fatal myocardial infarction, hospital admission for unstable angina, coronary or arterial revascularisation, fatal and non-fatal stroke, or transient ischaemic attack) and harm outcomes (type 2 diabetes, mild cognitive impairment, rhabdomyolysis, and myopathy)

Outcomes	Rate (95% CI) per 1000 person years		Hazard ratio(95% CI)
	Events in statin initiators	Events in non-initiators	
Cardiovascular disease	19.0 (17.8 to 20.4)	24.2 (23.1 to 25.3)	0.79 (0.72 to 0.87)
Diabetes	17.8 (16.3 to 19.4)	15.9 (15.3 to 16.4)	1.12 (1.01 to 1.25)
Cognitive impairment	1.8 (1.4 to 2.3)	1.6 (1.4 to 1.8)	1.13 (0.82 to 1.56)
Rhabdomyolysis	0.76 (0.52 to 0.95)	0.7 (0.5 to 0.9)	1.09 (0.68 to 1.75)
Myopathy	2.9 (2.4 to 3.4)	2.6 (2.3 to 3.1)	1.10 (0.76 to 1.62)

CI, confidence interval.

Statin initiators had a higher risk of diabetes (hazard ratio 1.12, 95% CI 1.01 to 1.25) than non-initiators. Other harm outcomes were rare (table 3), and starting statins had no significant effect on cognitive impairment (hazard ratio 1.13, 95% CI 0.82 to 1.56), myopathy (1.10, 0.76 to 1.61), or rhabdomyolysis (1.09, 0.68 to 1.75).

Analysis of specific statin types was restricted to cardiovascular disease and diabetes outcomes with sufficiently large sample sizes. The greatest reduction in the risk of cardiovascular disease was associated with rosuvastatin (hazard ratio 0.76, 95% CI 0.66 to 0.87) and atorvastatin (0.76, 0.69 to 0.84), followed by fluvastatin (0.80, 0.67 to 0.90) and pravastatin (0.86, 0.79 to 0.94). We found a lower risk of diabetes with pravastatin (hazard ratio 0.99, 95% CI 0.90 to 1.08) and fluvastatin (1.05, 0.83 to 1.35), and a slightly higher risk with rosuvastatin (1.13, 0.98 to 1.30) and atorvastatin (1.10, 1.00 to 1.23). Long term use (>12 months) also showed a slightly greater benefit (hazard ratio 0.76, 95% CI 0.68-0.84) but increased the risks of diabetes (1.16, 1.05 to 1.29). Random placebo test showed no effect on cardiovascular disease (hazard ratio 1.01, 95% CI 0.93 to 1.10) or diabetes (1.00, 0.90 to 1.11), with no indication of residual confounding.

**Figure 2:**
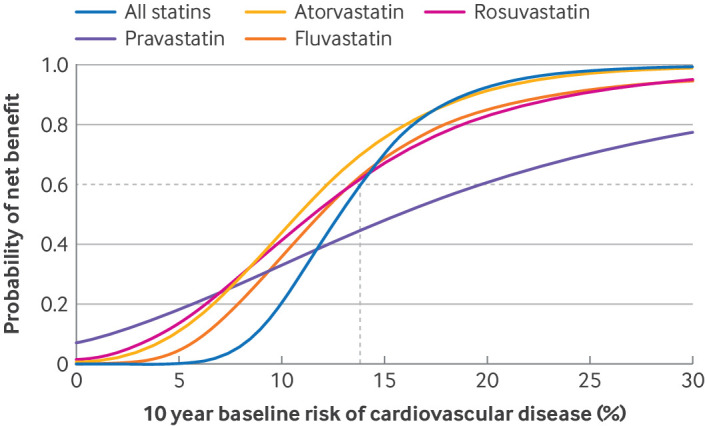
Distribution of benefit-harm balance across the baseline cardiovascular disease risk spectrum for all statins and for specific statins. Threshold for baseline cardiovascular disease risk, above which the prevention of cardiovascular disease outweighed the harmful outcomes, was 13.8% over 10 years (dotted lines)

Table 4 shows the input estimates for the benefit-harm analysis. The threshold for baseline risk of cardiovascular disease, above which the prevention of cardiovascular disease outweighed the harmful outcomes, was 13.8% over 10 years, on average (figure 2). The net benefit at the calculated threshold was 1.58 (instead of 0 as for the naive threshold calculations; 95% uncertainty interval 1.18 to 2.00) cardiovascular disease equivalent risk prevented per 1000 persons over 10 years (online supplemental figures S8 and S9). Online supplemental tables S4 and S5 show the 10 year cumulative risks of the outcomes, with and without statins. The benefit-harm balance varied according to personal characteristics and clinical subgroups (online supplemental tables S6 and S7). The risk thresholds also varied by statin type, with thresholds of 12.3% for atorvastatin, 13.5% for rosuvastatin, 13.4% for fluvastatin, and 19.4% for pravastatin (figure 2).

**Table 4:** Input estimates for the analysis of benefit-harm balance

Outcomes	Hazard ratio (95% CI)		
	Relative effect of statin treatment	Outcome rates in statin non-initiators (per 1000 person years)	Outcome preference weights‡^24^
Cardiovascular disease	0.79 (0.72 to 0.87)	10 year baseline risks 0-30%*	0.741 (0.686 to 0.800)
Diabetes	1.12 (1.01 to 1.25)	15.9 (15.3 to 16.4)	0.246 (0.209 to 0.290)
Cognitive impairment	1.13 (0.82 to 1.56)	1.6 (1.4 to 1.8)	0.100 (0.070 to 0.142)
Rhabdomyolysis	1.09 (0.68 to 1.75)	0.7 (0.5 to 0.9)	0.741 (0.686 to 0.800)†
Myopathy	1.10 (0.76 to 1.62)	2.6 (2.3 to 3.1)	0.100 (0.071 to 0.142)
Acute renal injury	1.12 (1.00 to 1.26)^7 22^	25.4 (24.7 to 26.1)	0.194 (0.165 to 0.247)
Cataracts	1.22 (1.03 to 1.49)^21 22^	2.1 (1.7 to 2.5)^20^	0.130 (0.108 to 0.170)
Liver dysfunction	1.40 (1.34 to 1.46)^7 22^	22.3 (21.6 to 22.9)	0.130 (0.108 to 0.170)

Data were obtained from the target trial, unless other sources are indicated.

* The benefit-harm evaluation was calculated for individuals with hypothetical 10 year baseline cardiovascular disease risks ranging from 0% to 30%.

† Rhabdomyolysis and cognitive impairment were not part of the preference study, and we considered them as important as cardiovascular disease and liver dysfunction, respectively.

‡ A preference of 0.0 corresponds to no concern, and 1.0 to the worst outcome.

CI, confidence interval.

In the sensitivity analyses, we examined how variations in individual preferences influenced the risk thresholds (online supplemental figure S10). Assigning hypothetical incremental preferences for cardiovascular disease from 0.50 to 1.00 lowered the threshold to start statins from 20.9% to 11.6% for 10 year cardiovascular disease risk. Similarly, the threshold varied from 12.5% to 25.5% when we assigned diabetes preferences from 0.0 to 1.00. Some outcomes were not sensitive to preference changes, however, because of their rare incidence and the lower treatment effects of statins. For example, an extreme preference change from 0 to 1 only resulted in a threshold change from 14.2% to 16.0% for cognitive impairment, 14.0% to 16.8% for myopathy, and 13.8% to 14.5% for rhabdomyolysis (online supplemental figure S10).

## Discussion

### Principal findings

We have provided comprehensive findings on the effectiveness, safety, and benefit-harm balance of different statins for primary prevention of cardiovascular disease in people with HIV using real world data. Patients who started statin treatment had 21% lower rates of cardiovascular disease events, but 12% higher rates of diabetes. The effect on stroke and myocardial infarction was 5% greater than the overall effect on cardiovascular disease.

On average, the benefits of statins exceeded the harms for individuals with a 10 year baseline risk of cardiovascular disease of ≥13.8%. However, the risk thresholds varied across individuals. Some individuals may be recommended statins at lower thresholds than the average, depending on their individual risk of harm outcomes and how they value the importance of cardiovascular disease relative to the potential harms. The risk thresholds also differed by statin type and clinical or patient phenotypes. Individuals with comorbidities, such as those with immunocompromised conditions, hypertension, hepatitis C virus infection, or older individuals, had higher risk thresholds (online supplemental table S7). This finding could be because of general frailty and increased risks for the harm outcomes of statins, which may offset the benefits of reduced cardiovascular disease events. We attempted to show the potential interactions of the effects of statins by subgroup characteristics that could add to the variations in risk thresholds in the subgroups (online supplemental table S3). These subgroup effects should be interpreted with caution, however, because the target trial design and analysis might not be sufficiently robust for subgroup estimations.

The variation in findings based on patient risks and preferences implies that use of one-size-fits-all thresholds could be overly simplistic. Starting treatment with statins should be customised to individual patients, taking into account their outcome risks and preferences. These individualised decisions can be facilitated by integrating the benefit-harm balance estimators into existing care procedures.

### Comparison with other studies

Our target trial findings were similar to those in the general population.^78 28^,^31^ REPRIEVE assessed only pitavastatin in prevention of cardiovascular disease in people with HIV,^10^ and our findings are broadly consistent with its results. Although qualitatively similar, REPRIEVE showed a greater reduction in the risk of cardiovascular disease from treatment with pitavastatin but also increased risks, particularly for diabetes and muscle disorders, compared with our target trial. Our study could be complementary to REPRIEVE by reflecting real world evidence.

Direct comparisons between our findings and REPRIEVE, however, should be made with caution. Statins differ in potency, which can influence effectiveness and safety outcomes.^29^ Although REPRIEVE tested pitavastatin, a glucuronidated statin known for its minimal risk of interaction with antiretroviral therapy,^2^ our analysis was of all statins (except lovastatin and simvastatin because of their higher risk of interactions).^2 13 18^ Pitavastatin was also part of our data but only contributed <1% of statin prescriptions, which shows its limited use in real world settings (online supplemental table S8).

Concerns inherent in observational studies might also explain the difference in estimates. Harm events, drug interactions, and increased comorbidity in real world practice could cause higher rates of early withdrawals or reduced adherence, subsequently diminishing the effects compared with controlled studies such as REPRIEVE. Treatments in real world settings might not be taken as recommended, affecting their effectiveness, compared with the setting of a randomised controlled trial. Lower doses of statins might often be used in practice to avoid the risk of drug interactions, which could compromise effectiveness, whereas participants in REPRIEVE took a fixed dose of 4 mg.^10^ Unlike REPRIEVE, which might have used modern and better quality antiretroviral treatments, our study used data collected over decades, including periods when low quality antiretroviral treatments were in use.^32^ The cardiovascular disease risk profiles of these treatments differ. These concerns, and the fact that we emulated a pragmatic, open label target trial (as opposed to the trial with blinding strategies) might collectively contribute to differences between REPRIEVE and our findings. Our results did not differ from those in studies of individuals without HIV,^78 28^,^31^ but different statins should be evaluated in controlled studies in the contemporary HIV population with current generations of antiretroviral therapy.

The findings of benefit-harm balance were similar to our previous study in the general population,^9^ but higher than those outlined in most guidelines, including those from the American College of Cardiology/American Heart Association, US Preventive Services Task Force, and European Society of Cardiology.^8 16 33^ The higher thresholds in our study are due to our quantitative assessment of the trade-off between benefits and harms on the same scale. This approach allows us to determine optimal risk thresholds where the reduction in the risk of cardiovascular disease started to exceed the cumulative harms. In contrast, most practices and guidelines use simplified metrics, such as number need to treat or number need to harm for each outcome in isolation, or their ratio,^34^ making determination of risk thresholds unclear. As a result, varying thresholds exist between guidelines, despite most being based on a similar body of evidence. We also accounted for preference together with outcome risks and treatment effects. Preference is an important factor that ultimately determines treatment decisions, but how this factor is included in clinical guidelines is often unclear.

### Strengths and limitations of this study

Our study provided a nuanced analysis of the effectiveness and safety of statins based on a target trial, as well as risk thresholds to guide decision making for statin treatment. The study also had some limitations which should be accounted for when interpreting the findings. We did not perform dose-effect analyses because of limited data on statin doses. Our assumption of adherence to statins might have been overstated. Substantial non-adherence because of harm outcomes, polypharmacy, drug interactions, and other reasons is possible. In a controlled study where adherence is measured and adjusted for, the effect of statins could be greater than what we found, as indicated by our explorative analyses limited to long term use of statins. This concern should be looked at in other data with adherence indicators.

Although we controlled for relevant factors or their proxies, unmeasured confounding variables might still exist, including diet, exercise, and excessive alcohol use. We mainly estimated intention-to-treat causal effects, and differential switching of treatments might have occurred because of changes in patient prognosis after enrolment in the target trial. Not all outcomes were evaluated in our target trial, and future studies should thus investigate all potential harm outcomes of statins in people with HIV.

We performed robustness checks for cardiovascular disease and diabetes that had sufficient data, but to a lesser extent for myopathy, rhabdomyolysis, and cognitive impairment because of the smaller number of events. Moreover, not all NA-ACCORD cohorts conducted outcome ascertainment and hence the benefit and harm outcomes cannot be guaranteed. The effectiveness and safety of statins on established benefit and harm outcomes, including death, should be evaluated in randomised controlled trials or larger international cohorts to ensure reproducibility of the effects. Moreover, although the NA-ACCORD cohorts were from broad geographical areas across the US and Canada, further research should look at the under-representation of some groups in the cohorts (eg, women, Asians), diverse populations, including low and middle income countries, and settings with different healthcare systems. We used preferences from sources other than NA-ACCORD, because studies indicate that measures of outcome specific preference or disability weights focusing on health loss do not vary across populations, unlike other broader constructs, such as quality adjusted life years or welfare loss measures.^23 35^ Our analysis, however, found that regardless of the sources from people with or wiithout HIV, aggregate preferences might not be relevant. These findings emphasise the need for customised decision making, taking into account individual patient preferences.

### Conclusions

Our study showed the potential benefit of statins as a class, and rosuvastatin, atorvastatin, pravastatin, and fluvastatin, for the primary prevention of cardiovascular disease events in people with HIV. Identifying a threshold at which subgroups or individual patients would derive more benefit than harm would be useful, based on a transparent and systematic approach. We provided global and grouped risk thresholds for starting statin treatment for different subgroups, but the analysis should be continuously updated and refined as more data accrues, such as data on the use of integrase strand transfer inhibitors and tenofovir alafenamide fumarate. To efficiently optimise the use of statins, decisions should be individualised by integrating a benefit-harm balance estimator, considering individual benefits, harms, and preferences, as well as the selection of antiretroviral therapy with minimal drug-drug interactions.

## Supplementary material

10.1136/bmjmed-2024-001132 Supplementary file 1

10.1136/bmjmed-2024-001132 Supplementary file 2

## Data Availability

Data are available upon reasonable request. Data may be obtained from a third party and are not publicly available.

## References

[1] Feinstein MJ, Hsue PY, Benjamin LA (2019). Characteristics, Prevention, and Management of Cardiovascular Disease in People Living With HIV: A Scientific Statement From the American Heart Association. Circulation.

[2] Waters DD, Hsue PY (2019). Lipid Abnormalities in Persons Living With HIV Infection. Can J Cardiol.

[3] Neesgaard B, Greenberg L, Miró JM (2022). Associations between integrase strand-transfer inhibitors and cardiovascular disease in people living with HIV: a multicentre prospective study from the RESPOND cohort consortium. Lancet HIV.

[4] Surial B, Mugglin C, Calmy A (2021). Weight and Metabolic Changes After Switching From Tenofovir Disoproxil Fumarate to Tenofovir Alafenamide in People Living With HIV : A Cohort Study. Ann Intern Med.

[5] Kileel EM, Malvestutto CD, Lo J (2023). Changes in Body Mass Index with Longer-term Integrase Inhibitor Use: A Longitudinal Analysis of Data from the Randomized Trial to Prevent Vascular Events in Human Immunodeficiency Virus (REPRIEVE). Clin Infect Dis.

[6] Fitch KV, Fulda ES, Grinspoon SK (2022). Statins for primary cardiovascular disease prevention among people with HIV: emergent directions. Curr Opin HIV AIDS.

[7] Yebyo HG, Aschmann HE, Kaufmann M (2019). Comparative effectiveness and safety of statins as a class and of specific statins for primary prevention of cardiovascular disease: A systematic review, meta-analysis, and network meta-analysis of randomized trials with 94,283 participants. Am Heart J.

[8] Mangione CM, Barry MJ, US Preventive Services Task Force (2022). Statin Use for the Primary Prevention of Cardiovascular Disease in Adults: US Preventive Services Task Force Recommendation Statement. JAMA.

[9] Yebyo HG, Aschmann HE, Puhan MA (2019). Finding the Balance Between Benefits and Harms When Using Statins for Primary Prevention of Cardiovascular Disease: A Modeling Study. Ann Intern Med.

[10] Grinspoon SK, Fitch KV, Zanni MV (2023). Pitavastatin to Prevent Cardiovascular Disease in HIV Infection. *N Engl J Med*.

[11] Sadeq A, Elnour AA, Farah FH (2023). A Systematic Review of Randomized Clinical Trials on the Efficacy and Safety of Pitavastatin. *Curr Rev Clin Exp Pharmacol*.

[12] Srichatrapimuk S, Wongsa A, Sungkanuparph S (2023). Effects of pitavastatin on atherosclerotic-associated inflammatory biomarkers in people living with HIV with dyslipidemia and receiving ritonavir-boosted atazanavir: a randomized, double-blind, crossover study. AIDS Res Ther.

[13] Lees RS, Lees AM (1995). Rhabdomyolysis from the coadministration of lovastatin and the antifungal agent itraconazole. *N Engl J Med*.

[14] Gange SJ, Kitahata MM, Saag MS (2007). Cohort profile: the North American AIDS Cohort Collaboration on Research and Design (NA-ACCORD). Int J Epidemiol.

[15] Hernán MA, Robins JM (2016). Using Big Data to Emulate a Target Trial When a Randomized Trial Is Not Available. Am J Epidemiol.

[16] Goff DC, Lloyd-Jones DM, Bennett G (2014). 2013 ACC/AHA guideline on the assessment of cardiovascular risk: a report of the American College of Cardiology/American Heart Association Task Force on Practice Guidelines. J Am Coll Cardiol.

[17] Grinspoon SK, Fitch KV, Overton ET (2019). Rationale and design of the Randomized Trial to Prevent Vascular Events in HIV (REPRIEVE). Am Heart J.

[18] Chastain DB, Stover KR, Riche DM (2017). Evidence-based review of statin use in patients with HIV on antiretroviral therapy. J Clin Transl Endocrinol.

[19] Stroes ES, Thompson PD, Corsini A (2015). Statin-associated muscle symptoms: impact on statin therapy-European Atherosclerosis Society Consensus Panel Statement on Assessment, Aetiology and Management. *Eur Heart J*.

[20] Rasmussen LD, Kessel L, Molander LD (2011). Risk of cataract surgery in HIV-infected individuals: a Danish Nationwide Population-based cohort study. Clin Infect Dis.

[21] Yusuf S, Bosch J, Dagenais G (2016). Cholesterol Lowering in Intermediate-Risk Persons without Cardiovascular Disease. N Engl J Med.

[22] Cai T, Abel L, Langford O (2021). Associations between statins and adverse events in primary prevention of cardiovascular disease: systematic review with pairwise, network, and dose-response meta-analyses. BMJ.

[23] Yebyo HG, Aschmann HE, Yu T (2018). Should statin guidelines consider patient preferences? Eliciting preferences of benefit and harm outcomes of statins for primary prevention of cardiovascular disease in the sub-Saharan African and European contexts. BMC Cardiovasc Disord.

[24] Gail MH, Costantino JP, Bryant J (1999). Weighing the risks and benefits of tamoxifen treatment for preventing breast cancer. J Natl Cancer Inst.

[25] Yebyo HG, Braun J, Menges D (2021). Personalising add-on treatment with inhaled corticosteroids in patients with chronic obstructive pulmonary disease: a benefit-harm modelling study. Lancet Digit Health.

[26] Arlegui H, Bollaerts K, Bauchau V (2020). Benefit-Risk Assessment of Vaccines. Part II: Proposal Towards Consolidated Standards of Reporting Quantitative Benefit-Risk Models Applied to Vaccines (BRIVAC). Drug Saf.

[27] von Elm E, Altman DG, Egger M (2007). The Strengthening the Reporting of Observational Studies in Epidemiology (STROBE) statement: guidelines for reporting observational studies. Ann Intern Med.

[28] Yu S, Jin J, Chen Z (2020). High-intensity statin therapy yields better outcomes in acute coronary syndrome patients: a meta-analysis involving 26,497 patients. Lipids Health Dis.

[29] Naci H, Brugts J, Ades T (2013). Comparative tolerability and harms of individual statins: a study-level network meta-analysis of 246 955 participants from 135 randomized, controlled trials. Circ Cardiovasc Qual Outcomes.

[30] Dickerman BA, García-Albéniz X, Logan RW (2019). Avoidable flaws in observational analyses: an application to statins and cancer. Nat Med.

[31] Dormuth CR, Filion KB, Paterson JM (2014). Higher potency statins and the risk of new diabetes: multicentre, observational study of administrative databases. BMJ.

[32] Tseng A, Seet J, Phillips EJ (2015). The evolution of three decades of antiretroviral therapy: challenges, triumphs and the promise of the future. Br J Clin Pharmacol.

[33] Visseren FLJ, Mach F, Smulders YM (2021). 2021 ESC Guidelines on cardiovascular disease prevention in clinical practice. Eur Heart J.

[34] Grundy SM, Stone NJ, Bailey AL (2019). 2018 AHA/ACC/AACVPR/AAPA/ABC/ACPM/ADA/AGS/APhA/ASPC/NLA/PCNA Guideline on the Management of Blood Cholesterol: A Report of the American College of Cardiology/American Heart Association Task Force on Clinical Practice Guidelines. Circulation.

[35] Salomon JA, Vos T, Hogan DR (2012). Common values in assessing health outcomes from disease and injury: disability weights measurement study for the Global Burden of Disease Study 2010. Lancet.

