# Correction: Statins for primary prevention of cardiovascular events in people with HIV: target trial and modelling study

**DOI:** 10.1136/bmjmed-2024-001132corr1

**Published:** 2025-07-07

 Yebyo HG, Guenthard HF, Rehfuess EA, *et al*. Statins for primary prevention of cardiovascular events in people with HIV: target trial and modelling study: *BMJ Med* 2025;4:e001132.

In this research article by Yebyo and colleagues (*BMJMED* 2025;4:e001132. doi: 10.1136/bmj-2024-001132, published 8 May), the second author's name has been corrected to Huldrych F Günthard.

