# Supplementary material for: Statins for primary prevention of cardiovascular events in people with HIV: target trial and modelling study
Source: BMJ Med. 2025 May 8;4(1):e001132. doi: 10.1136/bmjmed-2024-001132 (PMC12090529; doi:10.1136/bmjmed-2024-001132)
Supplement: Supplementary file 1 [file bmjmed-4-1-s001.pdf]

NA-ACCORD cohorts

There are several contributing cohorts to the NA-ACCORD database/ Each contributing cohort (listed below) submits data annually in a standardized format to the Data Management Core (DMC) at the University of Washington, Seattle, Washington, USA. After synchronizing and assessing the quality, the DMC transfers the data to Johns Hopkins University, where cohort-specific observation-window for outcomes are identified to minimize the risk of falsely assuming complete event ascertainment from electronic health records.[1]

All cohorts participating in the NA-ACCORD (and their principal investigators) are listed below. Cohorts contributing participants to our life expectancy estimates are in italics.

AIDS Link to the IntraVenous Experience (Gregory D. Kirk);

Adult AIDS Clinical Trials Group Longitudinal Linked Randomized Trials (Constance A. Benson, Ronald J. Bosch, and Ann C. Collier);

Fenway Health HIV Cohort (Stephen Boswell, Chris Grasso, Ken Mayer)

HAART Observational Medical Evaluation and Research (Robert S. Hogg, Richard Harrigan, Julio Montaner, Hasina Samji, and Angela Cescon);

HIV Outpatient Study (John T. Brooks and Kate Buchacz);

HIV Research Network (Kelly A. Gebo and Richard Moore);

Johns Hopkins HIV Clinical Cohort (Richard D. Moore);

John T. Carey Special Immunology Unit Patient Care and Research Database, Case Western Reserve University (Benigno Rodriguez);

Kaiser Permanente Mid-Atlantic States (Michael A. Horbert);

Kaiser Permanente Northern California (Michael A. Horberg, and Michael J. Silverberg);

Longitudinal Study of Ocular complications of AIDS (Jennifer E. Thorne);

Multicenter Hemophilia Cohort Study–II (James J. Goedert);

Multicenter AIDS Cohort Study (Lisa P. Jacobson);

Montreal Chest Institute Immunodeficiency Service Cohort (Marina B. Klein);

Ontario HIV Treatment Network Cohort Study (Sean B. Rourke, Ann Burchell, and Anita R. Rachlis);

Retrovirus Research Center, Puerto Rico (Robert F. Hunter-Mellado and Angel Mayor)

Southern Alberta Clinic Cohort (M. John Gill);

Studies of the Consequences of the Protease Inhibitor Era (Steven G. Deeks and Jeffery N. Martin);

University of Alabama at Birmingham 1917 Clinic Cohort (Michael S. Saag, Michael Mugavero, and James Willig);

University of North Carolina, Chapel Hill HIV Clinic Cohort (Joseph J. Eron, and Sonia Napravnik);

University of Washington HIV Cohort (Mari M. Kitahata and Heidi M. Crane);

Veterans Aging Cohort Study (Amy C. Justice, Robert Dubrow, and David Fiellin);

Vanderbilt-Meharry CFAR Cohort (Timothy R. Sterling, David Haas and Sam Stinnette);

Women's Interagency HIV Study (Stephen J. Gange and Kathryn Anastos).

NA-ACCORD Executive Committee

Richard D. Moore, Michael S. Saag, Stephen J.Gange, Mari M. Kitahata, Rosemary G. McKaig, Amy C. Justice and Aimee M. Freeman.

Epidemiology/Biostatistics Core

Stephen J. Gange, Alison G. Abraham, Bryan Lau, Keri N. Althoff, Jinbing Zhang, Jerry Jing, Elizabeth Golub, Shari Modur, David Hanna, Peter Rebeiro, Adell Mendes, and Cherise Wong.

Data Management Core

Mari M. Kitahata, Stephen E. Van Rompaey, Heidi M. Crane, Eric Webster, Liz Morton, and Brenda Simon.

Ethical Approval

The human subjects activities of the NA-ACCORD has been approved by the Johns Hopkins School of Medicine institutional review board (NA_00002683) as well as the local institutional review boards at each of the participating cohorts, as follows: Johns Hopkins Bloomberg School of Public Health Office for Research Subjects (ALIVE, MACS, WIHS), Harvard School of Public Health Human Subjects Administration (ALLRT), Providence Health Care Research Institute Office of Research Services (HOMER), Center for Disease Control Human Research Protection Office (HOPS), Johns Hopkins School of Medicine Office of Human Subjects Research (HIVRN, JHHCC), University Hospitals of Cleveland Institutional Review Board for Human Investigations (CWRU), Kaiser Foundation Research Institute Institutional Review Board (KPNC), National Cancer Institute Central Institutional Review Board (MHCS-II), McGill University Health Center Biomedical Research Ethics Board (MONT), University of Calgary Office of Medical Bioethics (SAC), University of California, San Francisco Office of Research (SCOPE), University of Alabama at Birmingham Institutional Review Board for Human Use (UAB), University of North Carolina at Chapel Hill Office of Human Research Ethics (UCHCC), University of Washington Office of Sponsored Programs, Human Subjects Division (UW), Vanderbilt University Institutional Review Board (VAND). All local cohorts have obtained written consent except the following: HOMER (Our IRB approves the retrospective use of anonymous administrative data without requiring consent. We provide an information sheet for participants in lieu of a consent form); KPNC (Our IRB provided a waiver of informed consent); MONT (Our IRB approves the anonymous use of data retrospectively abstracted from clinical care databases without requiring consent. Our patients sign a general waiver on opening a medical chart at the hospital but no specific study related consent); SAC (Written consent to treatment by all patients and use of anonymous administrative data approved by ethics); SCOPE (We were granted a waiver for informed consent by our IRB.); VAND (Patients included in our NA-ACCORD dataset do not provide informed consent. We use previously collected ("on the shelf") clinical data. We do have IRB approval for NA-ACCORD studies). The need for informed consent for NA-ACCORD, as a study, is waived because IRB review determined that our research does not involve human subjects' research under the DHHS or FDA regulations.

Table S1: Outcomes

Variable	Value	Included data	Exclusion
Diabetes	0/1	Patient had HgA1c >6.5% OR diabetes-specific medication OR diabetes diagnosis	Diabetes history before index date
Cardiovascular disease	0/1	Acute fatal and nonfatal myocardial infarction (MI)<br>Coronary artery disease, non- MI<br>Acute stroke<br>Cardiovascular mortality<br>Peripheral vascular disease<br>Transient ischemic attack (TIA)	Events before index date<br><br>Events with unspecified time frame
Acute kidney injury§	0/1	Acute kidney injury	Chronic kidney disease<br>Kidney disease unspecified<br>End stage renal disease<br>Chronic glomerulonephritis<br>Chronic nephropathy<br>Kidney Transplant<br>Renal dialysis hemodialysis<br>Renal dialysis peritoneal<br>Renal dialysis unspecified<br>Acute renal injury before index date
Liver dysfunction§	0/1	Higher than 3 times the upper limit of Aspartate transferase (AST) and Alanine transaminase (ALT)	Events in the last 2 months
Myopathy	0/1	Creatine kinase > 10 times the upper limit of the normal range	>40 times the upper limit of the normal range (such cases may be rhabdomyolysis, which was treated as a separate outcome)<br>Events in the last 2 months
Rhabdomyolysis	0/1	Rhabdomyolysis	Events in the last 6 months
Mild cognitive impairment	0/1	Mild cognitive impairment	HIV-dementia<br>Events before index date

§ Outcomes were not included in the target trial. However, we included them to estimate the baseline risks in the non-initiators as input for the benefit-harm balance model.

Figure S1: Covariate balance plot for cardiovascular disease

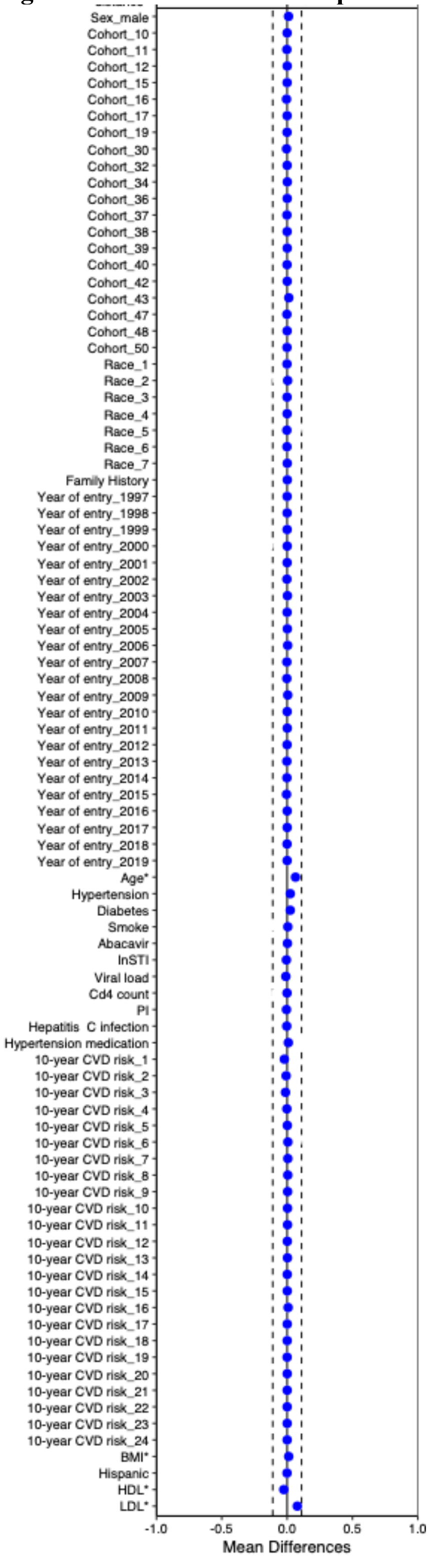

A covariate balancing plot showing the difference in standardized means of the variables used for matching. A balancing threshold of 0.1[2] was used to select the variables. * Indicates continuous variables for which mean differences were standardised.

Figure S2: Covariate balance plot for type 2 diabetes

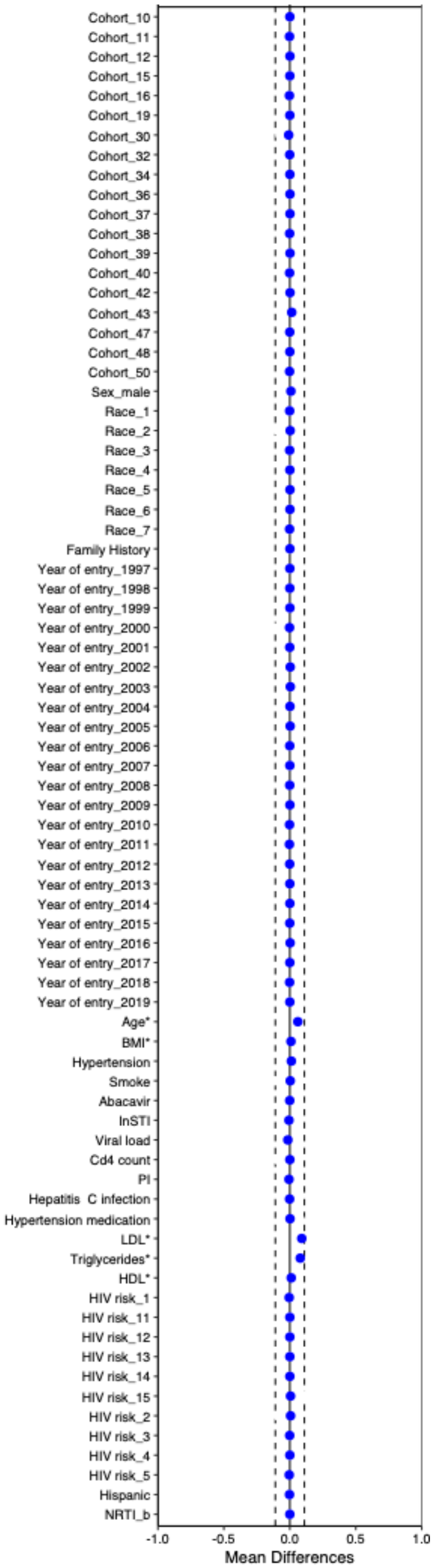

A covariate balancing plot showing the difference in standardized means of the variables used for matching. A balancing threshold of 0.1[2] was used to select the variables. * Indicates continuous variables for which mean differences were standardised.
2

Figure S3: Covariate balance plot for myopathy

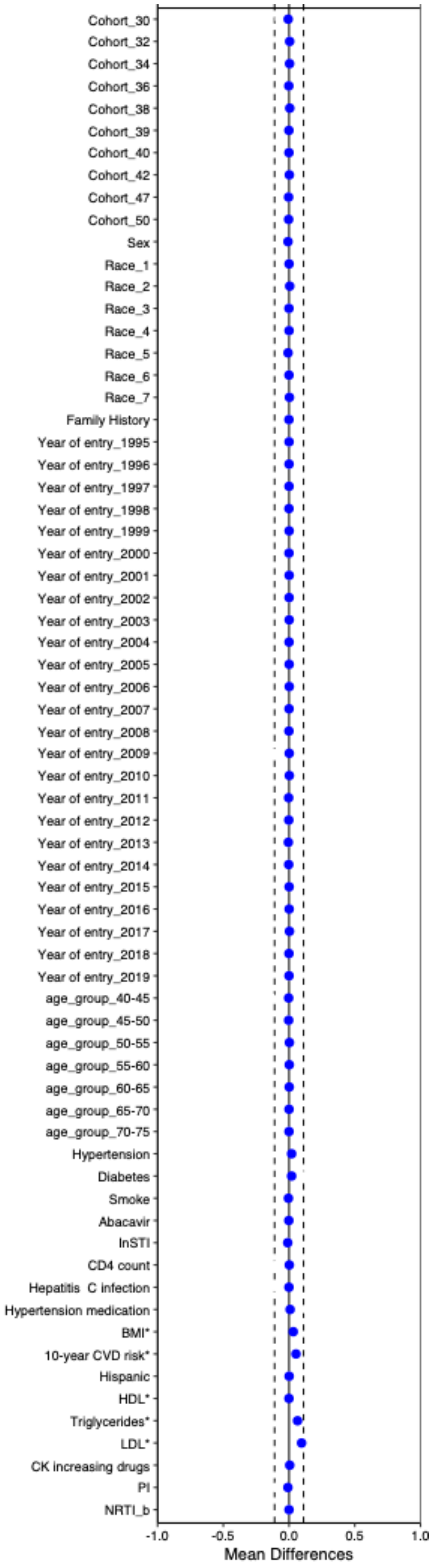

Figure S4: Covariate balance plot for mild cognitive impairment

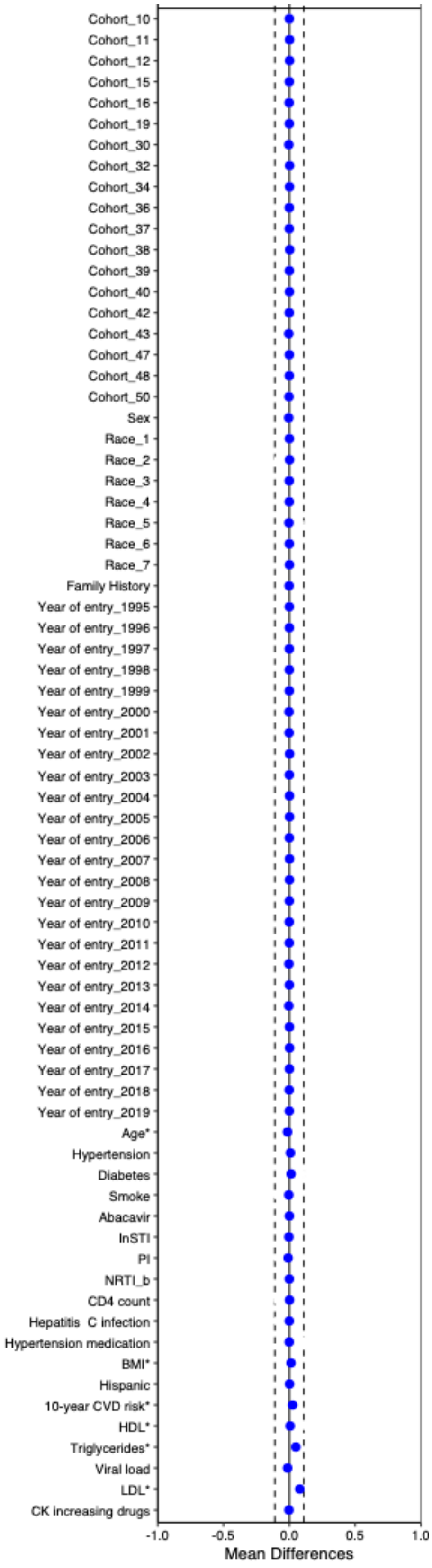

Figure S5: Covariate balance plot for rhabdomyolysis

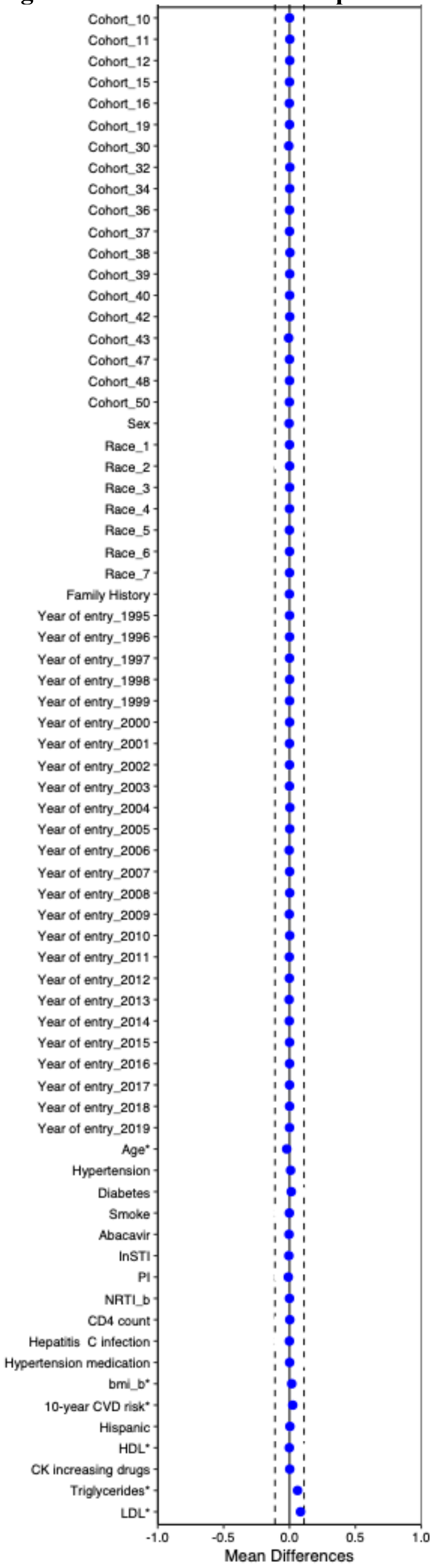

Predicting benefit and harm risks

To assess the 10-year net benefit of statins, we weighed the cumulative benefits (in terms of CVD risk reduction) against the cumulative outcomes of harms over the same period.[3–8] Our aim was to determine the baseline threshold for CVD risk, which would help identify individuals who would benefit the most from statins. To do this, we considered all individuals with a range of hypothetical baseline CVD risks (from 0 to 30%). For each level of risk, we predicted the CVD reduction with and without use of statins over a 10-year period using exponential model as follows:

1. Cumulative risk of experiencing benefit and harm events in statin non-initiators over a 10-year horizon was estimated as $p_i = \frac{I_i}{I_i+m} \times (1 - e^{-(I_i+m)\times 10\, years})$; where I_i is baseline risk of an outcome; m competing risk of death.
2. Cumulative risk of experiencing the benefit and harm outcomes over the 10-year horizon for PHW treated with statins was estimated, as $p_j = \frac{I_i \times rr_i}{I_i \times rr_i + m} \times (1 - e^{-(I_i \times rr_i + m)\times 10})$; where rr_i was relative effect of statins vs. no statins obtained from the target trial for most outcomes as well as from external sources for liver and renal dysfunction and cataract.
3. Risk differences of the benefit and harm outcomes were calculated from the above two estimations for a constant cohort of 1000 people, as $(p_i - p_j)\ \times 1000$.
4. To summarize the benefits and risks in a single number, we weighed the events by their respective preference values, as $(p_i - p_j) \times w_k$. where w was the preference weight towards the k outcomes. The preference is a measure of relative importance of the outcomes, which indicates patient's willingness to accept or risk-averse in pursuit of the benefit.
5. The risk differences adjusted by preference weights were aggregated to yield a single benefit-harm balance index (i.e., net clinical benefit), as $\sum_{k=1}^{n}(p_i - p_j) \times w_k$; where n was number of benefit and harm outcomes related to statin use. The index shows whether the averted CVD risks over 10 years outweighed the harms (positive index) or vice versa (negative index) or shows an equipoise (index equals zero).

The above process was performed for all possible baseline CVD risks from 0–30% over 10 years as well as baseline risk of harm outcomes. The analysis was done stochastically with 100,000 repetitions accounting for the statistical uncertainty of the input estimates (baseline risks, treatment effects, competing risk and preferences) to generate a distribution of the net clinical benefit. Interpreting the index can be challenging since it aggregates various outcomes. We provided a proxy interpretation by transforming the index to CVD-equivalent. Since we multiplied the risk differences by the respective preference weights (in step 5 above), this converted the index to CVD-equivalent, dividing the index by the preference weight of CVD, as $\frac{\sum_{k=1}^{n}(p_i - p_j)\times w_k}{w_{CVD}}$. This can be interpreted as the number of CVD-equivalent events averted by statins over 10 years without experiencing any worrisome harm outcomes.

For a clearer and more intuitive interpretation, we emphasized presenting the results in terms of the probability of net benefit. We calculated the probability that statin initiators would have more benefit than harm (net benefit)

compared with non-initiators from the benefit-harm index distribution. Statin therapy was considered to be net beneficial when the probability reached at least 0.60. The CVD baseline risk (across the 0 to 30% 10-year score) corresponding to the 0.60 probability of net benefit was defined as the risk threshold (see Appendix Figure 8 and 9). The point of equipoise (a 0.50 probability of net benefit) is a naïve threshold at which the average expected net benefit equals zero. But we chose 0.6 probability to define the threshold to ensure a non-zero minimal net benefit, since it would not be sensible for patients to initiate a treatment without any benefit, as happens when we take 0.5, where the net benefit is zero. However, this can be arguable, and we provide the threshold defined at the probability of 0.5 for those who want to take this definition (we provide additional explanation about the selection of 0.6 in appendix xx).

We additionally reported the expected net benefit, CVD (for each 0 to 30% baseline risk) as well as harm cumulative risks with and without statins over 10 years (see appendix x). The analyses included a bootstrapping with 1000 replicate samples to estimate 95% uncertainty intervals based on the 2.5th and 97.5th percentiles in the distributions of net benefit and outcome events. [9–12]

Figure S6: Flowchart for selection of eligible individuals from NA-ACCORD data when emulating the target trial

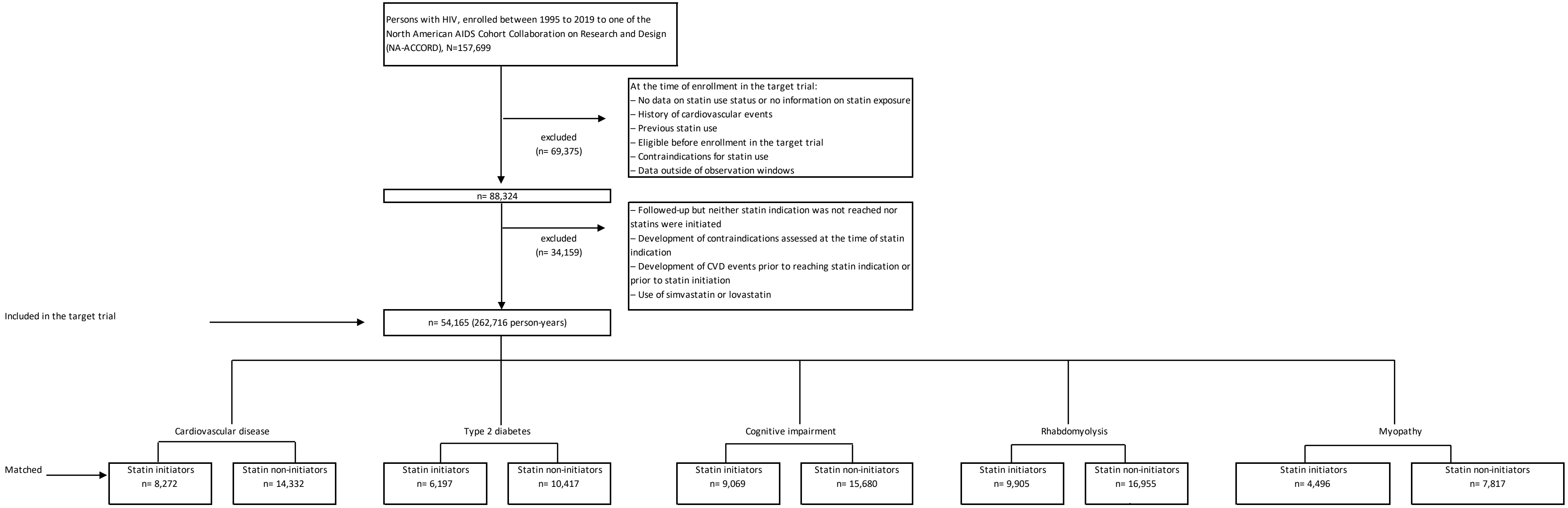

Table S2: Baseline characteristics of all participants in the target trial before matching

	Statin non-users (n=40662)	Statin users (n=13503)
**Sex (male), %**	35453 (87.2)	11866 (87.9)
**Race, %**		
Black	18441 (45.4)	5107 (37.8)
White	16174 (39.8)	6398 (47.4)
Asian	364 (0.9)	119 (0.9)
Others	5683 (14.0)	1879 (13.9)
**Ever smokers, %**	32464 (79.8)	9155 (67.8)
**Hypertension, %**	11009 (27.1)	6417 (47.5)
**Diabetes, %**	3108 (7.6)	2909 (21.5)
**Family history of CVD, %**	297 (0.7)	162 (1.2)
**NRTI use, %**	328 (0.8)	20 (0.1)
**Protease inhibitors use, %**	4893 (12.0)	1018 (7.5)
**InSTI use, %**	2805 (6.9)	1280 (9.5)
**Hepatitis C infection, %**	3125 (7.7)	155 (1.1)
**Hypertension medication, %**	6475 (15.9)	3598 (26.6)
**Abacavir use, %**	3150 (7.7)	1262 (9.3)
**HIV acquisition risk , %**		
Injection drug use	9448 (23.2)	3225 (23.9)
Men to men homosexual	10412 (25.6)	2050 (15.2)
Heterosexual	6328 (15.6)	1872 (13.9)
Unknown or others (hemophilia, blood transfusion, perinatal)	14474 (35.6)	6356 (47.1)
**Year of enrolment in the target trial, %**		
1995 to 1999	672 (1.7)	7 (0.1)
2000 to 2005	13489 (33.2)	2129 (15.8)
2006 to 2012	8846 (21.8)	2935 (21.7)
2013 to 2019	17655 (43.4)	8432 (62.4)
**Body mass index, %**		
<25	22062 (54.3)	4318 (32.0)
25 to <30	12468 (30.7)	5356 (39.7)
≥30	6132 (15.1)	3829 (28.4)
**Age group, %**		
≥40 to 65	39106 (96.2)	12291 (91.0)
≥65 to 75	1556 (3.8)	1212 (9.0)
**CD4**		
<200 cells/mm3	7093 (17.4)	1193 (8.8)
≥200 cells/mm3		
**Mean total cholesterol (mg/dL), sd**	166 (40)	223 (51)
**Mean LDL cholesterol (mg/dL), sd**	91 (31)	134 (42)
**Mean HDL cholesterol (mg/dL), sd**	36 (15)	45 (14)
**Systolic blood pressure, sd**	115 (14)	128 (9)
**Mean BMI, sd**	25 (5)	28 (6)
**Mean age, sd**	49 (7)	53 (8)
**10-year CVD risk score, sd**	8 (4)	15 (9)

Figure S7: 10-year cumulative incidence of cardiovascular disease and harm outcome events of statin initiators and non-initiators

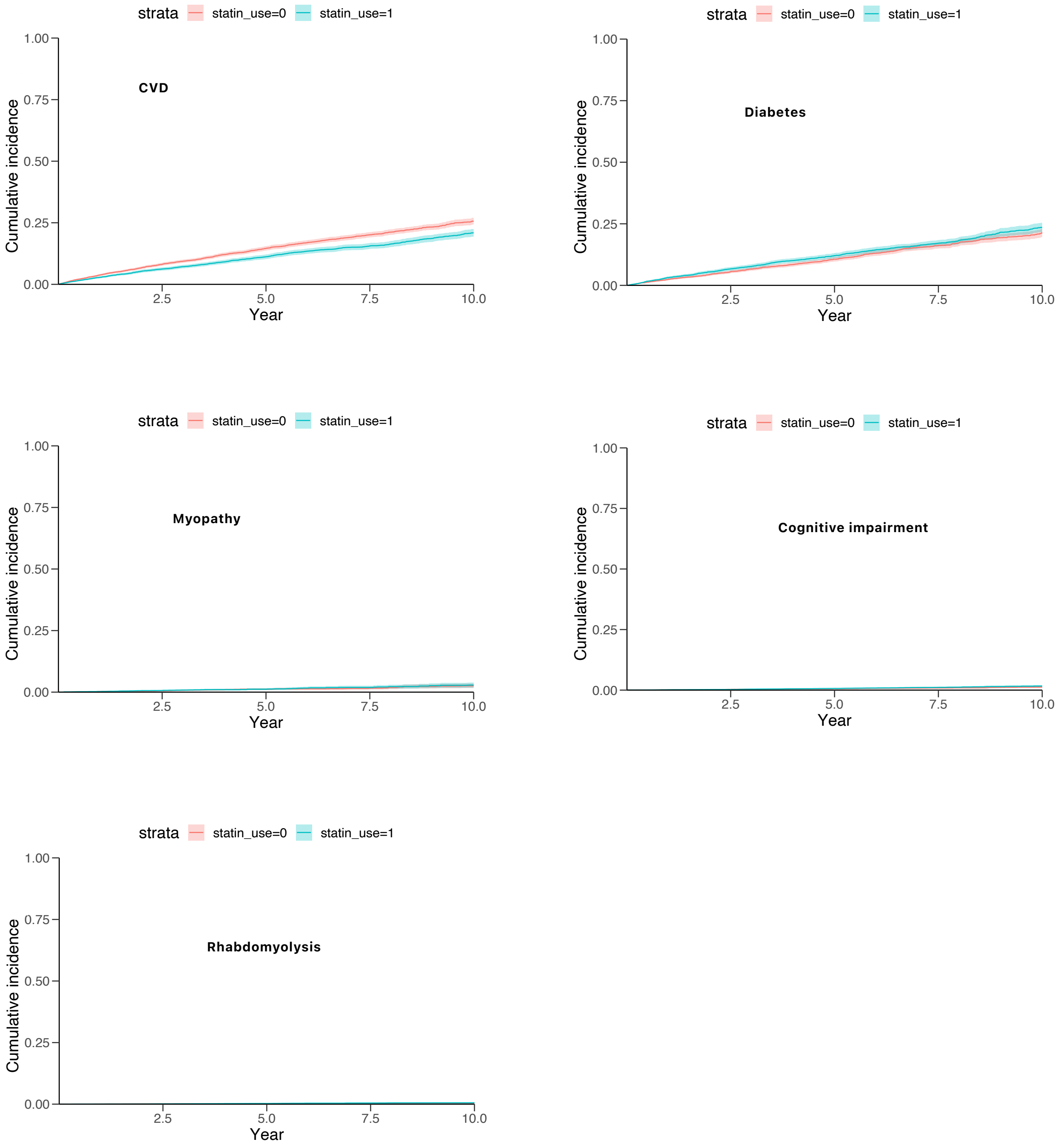

Table S3: Subgroup effects of statins on CVD and diabetes

	Subgroups	Cardiovascular events<br>HR (95% CI)	Type 2 diabetes<br><br>HR (95% CI)
Sex	Women	0.89 (0.65, 1.21)	1.34 (0.97, 1.85)
	Men	0.78 (0.71, 0.86)	1.10 (0.72, 1.24)
Diabetes	Yes	0.57 (0.47, 0.70)	NA
	No	0.83 (0.75, 0.93)	1.12 (1.01, 1.25)
Hypertension	Yes	0.72 (0.62, 0.83)	1.00 (0.83, 1.17)
	No	0.82 (0.72, 0.92)	1.27 (1.09, 1.49)
CD4 count	<200 cells/mm^3	0.84 (0.64, 1.12)	1.44 (1.01, 2.06)
	≥200 cells/mm^3	0.79 (0.72, 0.87)	1.10 (0.72, 1.24)
Age	40-60	0.79 (0.71, 0.88)	1.26 (1.11, 1.44)
	>=60-70	0.76 (0.61, 0.95)	0.80 (0.53, 1.21)
InSTI	Yes	1.05 (0.64, 1.74)	1.15 (0.74, 1.78)
	No	0.80 (0.73, 0.87)	1.13 (1.00, 1.27)
HCV	Yes	0.68 (0.36, 1.28)	NE
	No	0.80 (0.73, 0.88)	1.15 (1.03, 1.30)
10-year CVD risk, %	<7.5%	0.84 (0.70, 1.00)	1.39 (1.18, 1.63)
	≥7.5 to 10%	0.75 (0.56, 0.95)	0.91 (0.69, 1.20)
	>10 to 15%	0.56 (0.47, 0.68)	1.00 (0.78, 1.25)
	≥ 15%	0.65 (0.55–0.77)	0.89 (0.69, 1.16)

NE: Not estimable due to fewer subgroup sample

Figure S8: The distribution absolute and probability of net benefit

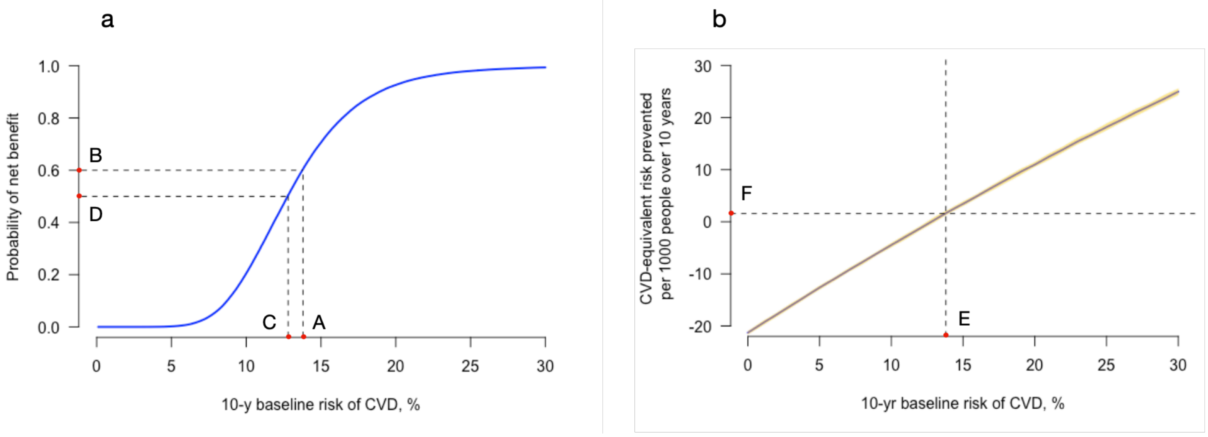

(a) The figure shows the probability of net benefit and (b) absolute net benefit

Point ***·A*** in plot (a) shows the 10-year CVD risk threshold (13.8%) to achieve more benefit than harm defined at a probability of 0.6 (indicated by ***·B***). Points ***·C*** a threshold of 12.8 10-year risk with a probability of 0.5 (indicated by***·D***). The net benefit at the threshold (13.8% baseline risk indicated by ***·E*** of plot b) is 1.58 CVD-equivalent risk avoided by statins without experiencing any harm outcomes. The net benefit when we used a 0.5 probability for defining a net benefit would apparently be zero.

Probability of 0.5 is a naive threshold for equipoise between benefits and harms. However, we cannot expect patients to take a long-term statin treatment if the expected net benefit is zero. We chose 0.6 to define CVD risk threshold to ensure that the benefits outweigh the harms. This will slightly raise the CVD risk threshold for initiating statin therapy. However, the selection of 0.6 can be considered arbitrary although it has been commonly used in many of our previous studies.[3–9] However, the selection of 0.6 over 0.5 probability does not make significant difference to the thresholds because of the nonlinear prosperity of the relationships between net benefit probability and baseline CVD risks, as shown in the figure below (i.e., there is less change in the 10-year CVD risk for every change in probability of net benefit). For example, the threshold for a 0.5 probability was a 12.8% 10-year risk, compared to a 13.8% 10-year risk for a 0.6 probability. In terms of absolute effects, at a probability of 0.5, the net benefit was zero. However, with a probability of 0.6, there was a benefit of 1.58 avoided CVD-equivalent events per 1,000 people over 10 years.

Figure S9: Distribution of benefit-harm index for 0–30% 10-year CVD risk

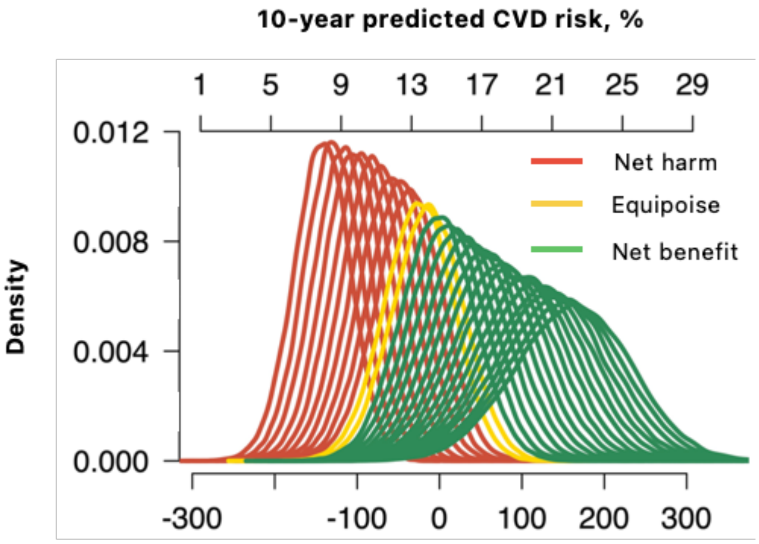

Distribution of benefit-harm index for 0 to 30% 10-year CVD risk

The distribution shows the preference-weighted benefit-harm balance for all negative and positive outcomes. It was calculated for all hypothetical 10-year baseline CVD risks from 0% to 30%. For baseline CVD risks <11.9%, the mean benefit-harm index showed more harms than benefits (red density curves), with a probability of net benefit <0.4. For baseline risks of 11.9 to <13.8%, the benefit-harm balance showed uncertainty about the net benefit (yellow density curves), as the probability of net benefit was around the equipoise (probabilities 0.4–0.6). For 10-year baseline risks of ≥13.8%, the average index showed more benefits than harms, with probability of net benefit ≥0.6 (green density curves). The dispersion of the distributions increased with increasing baseline CVD risk.

Table S4: 10-year cumulative CVD risks with and without statin use

10-year baseline CVD risk	Benefit-harm index in terms of CVD-equivalent risks	Probability of net benefit	10-year cumulative CVD risk with statin use	10-year cumulative CVD risk without statin use¶
0.0%	-21.25 (-21.52, -20.97)	0	0 (0, 0)	0 (0, 0)
1.0%	-19.51 (-19.79, -19.24)	0	7 (6, 7)	8 (8, 8)
2.0%	-17.79 (-18.08, -17.49)	0	13 (12, 14)	17 (17, 17)
3.0%	-16.07 (-16.33, -15.79)	0	20 (18, 22)	25 (25, 25)
4.0%	-14.38 (-14.68, -14.1)	0.001	26 (24, 29)	33 (33, 33)
5.0%	-12.69 (-12.97, -12.39)	0.003	33 (30, 36)	42 (42, 42)
6.0%	-11.01 (-11.33, -10.69)	0.01	40 (36, 44)	50 (50, 50)
7.0%	-9.35 (-9.66, -9.03)	0.028	47 (42, 51)	58 (58, 59)
8.0%	-7.7 (-8, -7.38)	0.065	53 (49, 58)	67 (67, 67)
9.0%	-6.06 (-6.41, -5.73)	0.126	60 (55, 66)	75 (75, 75)
10.0%	-4.45 (-4.76, -4.13)	0.21	67 (61, 73)	84 (83, 84)
11.0%	-2.83 (-3.19, -2.45)	0.312	74 (67, 80)	92 (92, 92)
12.0%	-1.23 (-1.59, -0.88)	0.421	80 (73, 88)	100 (100, 101)
13.0%	0.35 (-0.03, 0.74)	0.528	87 (80, 95)	109 (108, 109)
**13.8%†**	**1.58 (1.18, 2)**	**0.606**	**93 (85, 101)**	**115 (115, 116)**
14.0%	1.9 (1.48, 2.32)	0.624	94 (86, 103)	117 (117, 118)
15.0%	3.47 (3.07, 3.87)	0.707	101 (92, 110)	126 (125, 126)

16.0%	5.01 (4.59, 5.44)	0.775	108 (98, 118)	134 (134, 134)
17.0%	6.53 (6.11, 6.95)	0.829	115 (105, 125)	142 (142, 143)
18.0%	8.04 (7.61, 8.45)	0.87	122 (111, 133)	151 (150, 151)
19.0%	9.54 (9.07, 9.97)	0.901	129 (118, 140)	159 (159, 160)
20.0%	11.03 (10.57, 11.51)	0.926	135 (124, 148)	168 (167, 168)
21.0%	12.5 (11.99, 12.99)	0.943	142 (130, 155)	176 (176, 177)
22.0%	13.92 (13.44, 14.46)	0.956	149 (137, 163)	185 (184, 185)
23.0%	15.37 (14.87, 15.84)	0.966	157 (143, 170)	193 (192, 194)
24.0%	16.8 (16.26, 17.35)	0.974	164 (150, 178)	202 (201, 202)
25.0%	18.19 (17.64, 18.76)	0.979	171 (157, 186)	210 (209, 211)
26.0%	19.57 (19.03, 20.12)	0.983	178 (163, 193)	219 (218, 219)
27.0%	20.92 (20.36, 21.48)	0.987	185 (170, 201)	227 (226, 228)
28.0%	22.3 (21.72, 22.91)	0.99	192 (176, 209)	236 (235, 236)
29.0%	23.62 (23.03, 24.26)	0.992	199 (183, 216)	244 (243, 245)
30.0%	24.94 (24.34, 25.53)	0.993	206 (190, 224)	253 (252, 254)

We have presented a sample of baseline CVD risk thresholds. However, we conducted the benefit-harm analysis for every 0.1% increase in the range of 0% to 30% 10-year risk.

¶ Little or no variation is observed in the predicted risk of CVD without statin use because we utilized hypothetical and exact baseline risks ranging from 0% to 30%. This limited variation is attributed to the statistical uncertainty associated with the competing risk of death.

† The CVD risk threshold.

Table S5: 10-year cumulative harm risks with and without statin use

Statin use	Diabetes	Cognitive impairment	Liver dysfunction	Renal dysfunction	Myopathy	Rhabdomyolysis	Cataract
Yes	137 (123, 151)	15 (11, 21)	225 (215, 236)	208 (184, 234)	25 (16, 36)	6 (4, 10)	21 (17, 25)
No	123 (119, 127)	13 (12, 15)	168 (163, 172)	188 (184, 193)	22 (19, 25)	6 (5, 7)	17 (14, 21)

We have one predicted event estimate for each treatment groups because, unlike CVD for which we predicted cumulative risk for every baseline risk in the range of 0% to 30%, we used the average risk of the harm outcomes in the benefit-harm analysis (see paper)

Table S6. Annual incidence of harm outcomes per 1,000 person-years in individuals not treated with statins, by subgroups.

	Subgroup	Type 2 diabetes	Cognitive impairment	Liver dysfunction	Acute renal failure	Myopathy	Rhabdomyolysis
Sex	Women	19.1 (17.5, 20.9)	1.0 (0.7, 1.5)	12.2 (10.9, 13.6)	21.4 (19.8, 23.3)	1.4 (0.9, 2.1)	0.3 (0.2, 0.6)
	Men	15.4 (14.8, 16.0)	1.7 (1.5, 1.9)	23.8 (2.31, 24.6)	26.0 (25.2, 26.8)	3.1 (2.6, 3.6)	0.8 (0.7, 0.9)
Diabetes	No	15.9 (15.3, 16.4)	1.6 (1.4, 1.8)	22.1 (21.4, 22.8)	24.5 (23.8, 25.2)	2.7 (2.3, 3.1)	0.7 (0.5, 0.8)
Hypertension	No	13.1 (12.5, 13.7)	1.7 (1.5, 1.9)	22.0 (21.2, 22.8)	25.4 (24.6, 26.2)	2.7 (2.3, 3.1)	0.7 (0.6, 0.8)
	Yes	24.8 (23.4, 26.3)	1.4 (1.1, 1.7)	23.1 (21.8, 24.4)	25.3 (24.0, 26.8)	2.7 (1.9, 3.8)	0.6 (0.4, 0.8)
Age	≥40 to 65	15.5 (14.9, 16.1)	1.6 (1.4, 1.7)	22.7 (22.0, 23.4)	25.1 (24.4, 25.8)	2.6 (2.3, 3.1)	0.7 (0.6, 0.8)
	≥65 to 75	26.3 (22.7, 30.5)	2.4 (1.5, 3.8)	17.0 (15.0, 19.3)	32.6 (28.6, 37.1)	4.8 (2.0, 11.5)	0.7 (0.3, 1.7)
CD4 count (cells/mm^3)	≥100	15.9 (15.3, 16.5)	1.6 (1.4, 1.8)	21.8 (21.0, 22.6)	24.6 (23.8, 25.3)	2.5 (2.1, 3.0)	0.6 (0.5, 0.8)
	<100	15.7 (14.3, 17.1)	1.6 (1.2, 2.1)	26.4 (24.4, 28.6)	29.7 (27.8, 31.6)	3.3 (2.5, 4.5)	0.8 (0.6, 1.2)
Protease inhibitor use	No	16.1 (15.5, 16.7)	1.6 (1.4, 1.8)	23.1 (22.3, 23.8)	24.7 (23.9, 25.4)	2.7 (2.3, 3.2)	0.7 (0.5, 0.8)
	Yes	14.6 (13.2, 16.0)	1.7 (1.3, 2.3)	22.4 (20.7, 24.2)	29.2 (27.3, 31.1)	2.5 (1.8, 3.5)	0.7 (0.5, 1.1)
InSTI use	No	15.7 (15.1, 16.3)	1.5 (1.3, 1.7)	23.0 (22.3, 23.8)	24.6 (23.9, 25.3)	2.7 (2.3, 3.2)	0.6 (0.5, 0.8)
	Yes	21.2 (18.0, 25.0)	3.9 (2.7, 5.6)	20.3 (17.1, 24.1)	47.1 (42.4, 52.4)	2.0 (1.0, 3.8)	1.3 (0.7, 2.4)
BMI	<25	11.4 (10.7, 12.1)	1.6 (1.4, 1.9)	23.3 (22.2, 24.5)	27.7 (26.7, 28.8)	2.7 (2.2, 3.3)	0.7 (0.5, 0.9)
	>=25 30	15.9 (14.9, 16.9)	1.4 (1.2, 1.7)	21.6 (20.4, 22.9)	22.8 (21.7, 24.0)	2.6 (2.0, 3.3)	0.6 (0.5, 0.9)
	>=30	32.3 (30.2, 34.6)	1.8 (1.4, 2.3)	18.1 (16.4, 19.8)	23.6 (22.0, 25.3)	2.7 (1.9, 3.9)	0.6 (0.4, 1.0)
Ever smoking	No	15.0 (13.9, 16.2)	2.1 (1.7, 2.6)	19.2 (18.0, 20.5)	21.3 (20.0, 22.7)	2.5 (1.7, 3.7)	0.5 (0.3, 0.7)
	Yes	16.1 (15.5, 16.8)	1.4 (1.3, 1.6)	24.4 (23.5, 25.3)	26.6 (25.8, 27.4)	2.7 (2.3, 3.2)	0.7 (0.6, 0.9)
Race	Black	18.1 (17.2, 19.0)	1.4 (1.2, 1.6)	21.3 (20.3, 22.2)	27.5 (26.5, 28.6)	3.0 (2.4, 3.6)	0.8 (0.6, 1.0)
	White	13.1 (12.3, 14.0)	1.7 (1.4, 2.0)	23.1 (22.1, 24.2)	23.1 (22.0, 24.1)	2.4 (1.9, 3.1)	0.7 (0.5, 0.9)
	Others	16.2 (14.7, 17.8)	2.0 (1.5, 2.5)	23.2 (21.5, 25.1)	24.8 (23.0, 26.7)	2.4 (1.6, 3.6)	0.3 (0.2, 0.6)
Hepatitis C infection	No	15.7 (15.1, 16.3)	1.6 (1.4, 1.8)	20.7 (20.0, 21.3)	24.7 (23.9, 25.4)	2.5 (2.1, 2.9)	0.6 (0.5, 0.7)
	Yes	17.6 (15.8, 19.7)	1.5 (1.1, 2.2)	40.9 (38.0, 44.0)	32.0 (29.6, 34.6)	4.3 (3.0, 6.1)	1.1 (0.7, 1.7)

Table S7. 10-year CVD risk thresholds above which statins provided more benefit than harm, by subgroups.

Characteristics	Subgroups	10-year baseline CVD risk threshold (%) determined at which the probability of benefit outweighing harm was 0.6	10-year baseline CVD risk threshold (%) determined at which the probability of benefit outweighing harm was 0.5
Overall		13·8	12·8
Sex	Women	11·1	10·3
	Men	14·2	13·2
Hypertension	No	13·3	12·3
	Yes	15·4	14·3
Age	40 to 64	13·8	12·8
	65 to 75	15·1	14·0
Viral load (cp/ml)	>200	13·6	12·6
	≤200	15·3	14·2
CD4 counts (cells/mm^3)	≥200	13·6	12·6
	<200	15·3	14·1
Protease inhibitor use	No	13·9	13·0
	Yes	14·0	13·0
Integrase strand transfer inhibitors use	No	13·9	12·9
	Yes	16·1	14·9
Body mass index (kg/m^2)	<25	13·6	12·6
	25 to 30	13·3	12·4
	≥30	14·9	13·8
Ever smoking	No	12·5	11·6
	Yes	14·5	13·4
Race	Black	14·2	13·1
	White	13·3	13·0
	Others	14·0	12·3
Hepatitis C infection	No	13·3	13·1
	Yes	18·6	17·0

The threshold was the 10-year baseline CVD risk above which absolute CVD risk reduction exceeded harms over 10 years.
Thresholds were defined at a predicted CVD risk level above which the probability of benefit outweighing the harms was (a) 0.6 and (b) 0.5.

It crucial to carefully interpret these subgroup thresholds. For example, PWH aged 65–75 had higher thresholds than those aged 40–64, and this was due to the age-wise increase of harm outcomes penalizing the benefit. But that does not mean that statins are more beneficial for younger people than older people; in fact, in most cases, the opposite is true. Older individuals would have higher CVD risk scores due to age and related factors, making them more likely to exceed the calculated age-specific threshold. Some guidelines, such as ESC [10], also show an increase in thresholds as a function of age. Similarly, the higher threshold in people with hypertension than without seems counterintuitive. However, people with hypertension are more sicker and often at higher risk of statin-related harms, such as diabetes and eye problems, which offset the benefits of statins and lead to higher risk thresholds. However, as with age, because hypertension is a CVD predictor, individuals with hypertension are usually more likely to meet the calculated minimum threshold to initiate statins compared to those without hypertension.

Figure S10: Influence of outcome preferences on CVD risk thresholds

Varying preference of benefit and harm outcome preferences ranging from 0.0 (0.25 for CVD) to 1.0 for each 5% increase.

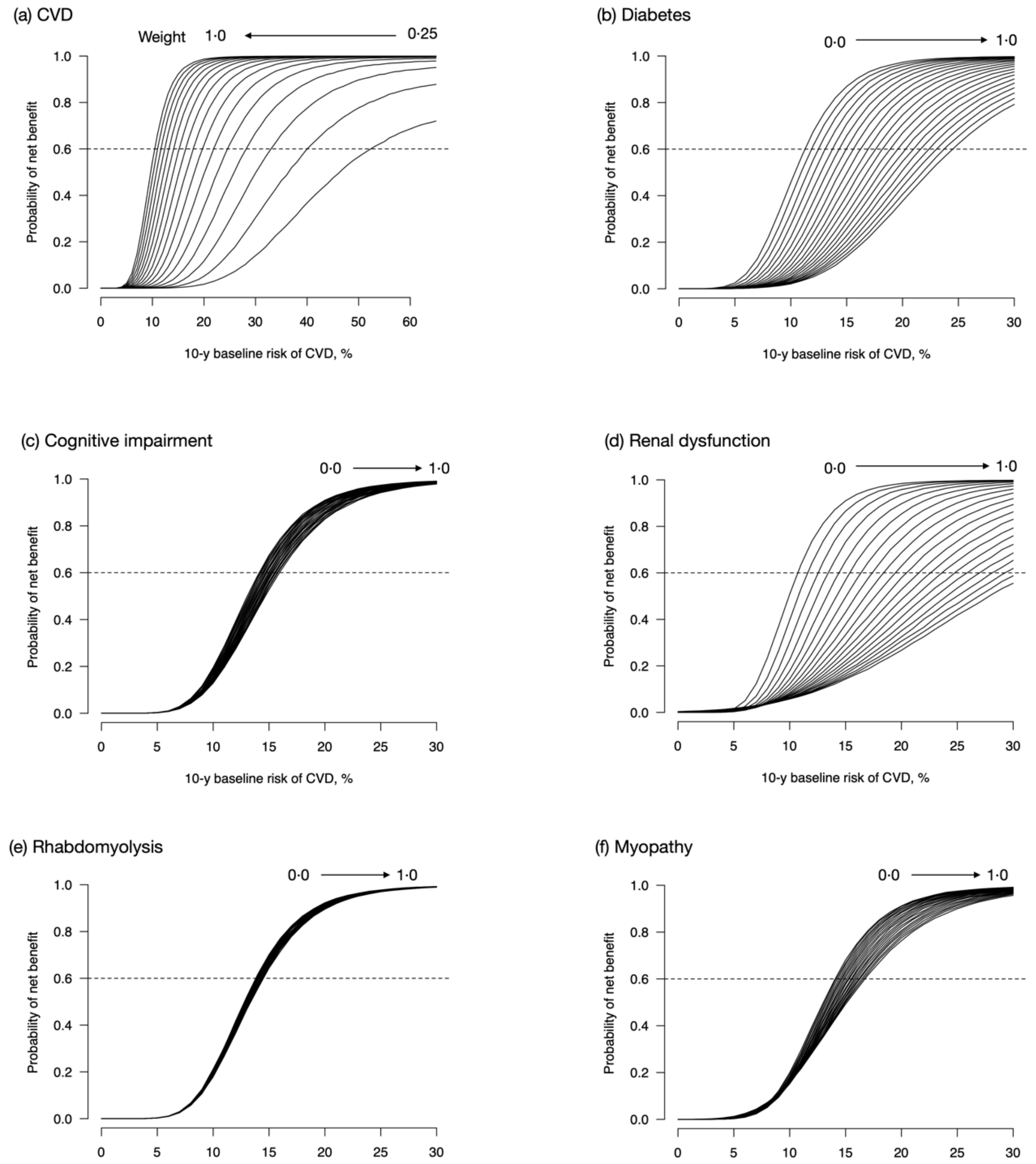

Table S8: Prescription rate of statins in NA-ACCORD data, 1995 to 2019

**Statin types**	**Overall prescription s**	**% of prescriptions**
Atorvastatin	90637	28.00
Cerivastatin	31	0.01
Fluvastatin	12810	4.00
Lovastatin	4976	1.56
Pitavastatin	240	0.075
Pravastatin	111021	34.89
Rosuvastatin	40900	12.85
Simvastatin	57565	18.09

References

1 Lang R, Hogan B, Zhu J, *et al.* The prevalence of mental health disorders in people with HIV and the effects on the HIV care continuum. *AIDS* 2023; **37**: 259–69.

2 Harder VS, Stuart EA, Anthony JC. Propensity score techniques and the assessment of measured covariate balance to test causal associations in psychological research. *Psychol Methods* 2010; **15**: 234–49.

3 Yebyo HG, Aschmann HE, Puhan MA. Finding the Balance Between Benefits and Harms When Using Statins for Primary Prevention of Cardiovascular Disease. *Ann Intern Med* 2018; **170**: 1.

4 Yebyo HG, Schmann HE, Menges D, Boyd CM, Puhan MA. Net benefit of statins for primary prevention of cardiovascular disease in people 75 years or older: a benefit–harm balance modeling study. *Ther Adv Vaccines* 2018; **9**: 259–61.

5 Puhan MA, Yu T, Stegeman I, Varadhan R, Singh S, Boyd CM. Benefit-harm analysis and charts for individualized and preference-sensitive prevention: Example of low dose aspirin for primary prevention of cardiovascular disease and cancer. *BMC Med* 2015; **13**: 1–11.

6 Yu T, Fain K, Boyd CM, *et al.* Benefits and harms of roflumilast in moderate to severe COPD. *Thorax* 2014; **69**: 616–22.

7 Yebyo HG, Braun J, Menges D, ter Riet G, Sadatsafavi M, Puhan MA. Personalising add-on treatment with inhaled corticosteroids in patients with chronic obstructive pulmonary disease: a benefit–harm modelling study. *Lancet Digit Health* 2021; **7500**: 1–10.

8 Gail MH, Costantino JP, Bryant J, *et al.* Weighing the risks and benefits of tamoxifen treatment for preventing breast cancer. *J Natl Cancer Inst* 1999; **91**: 1829–46.

9 Aschmann HE, Boyd CM, Robbins CW, *et al.* Balance of benefits and harms of different blood pressure targets in people with multiple chronic conditions: A quantitative benefit-harm assessment. *BMJ Open* 2019; **9**. DOI:10.1136/bmjopen-2018-028438.

10 Visseren FLJ, MacH F, Smulders YM, *et al.* 2021 ESC Guidelines on cardiovascular disease prevention in clinical practice. Eur Heart J. 2021; **42**: 3227–337.
