# Supplementary material for: Statins for primary prevention of cardiovascular events in people with HIV: target trial and modelling study
Source: BMJ Med. 2025 May 8;4(1):e001132. doi: 10.1136/bmjmed-2024-001132 (PMC12090529; doi:10.1136/bmjmed-2024-001132)
Supplement: Supplementary file 3 [file bmjmed-2024-001132.reviewer_comments.pdf]

PEER REVIEW HISTORY

BMJ Medicine publishes all reviews undertaken for accepted manuscripts. Reviewers are asked to complete a checklist review form and are provided with free text boxes to elaborate on their assessment. These free text comments are reproduced below.

ARTICLE DETAILS

**TITLE (PROVISIONAL)**	Statins for primary prevention of cardiovascular events in persons with HIV: A target trial and modelling study
**AUTHORS**	Yebyo, Henock; Guenthard, Huldrych; Rehfuess, Eva; Serra, Nicola; Haile, Sarah; Senn, Oliver; Lucas, Gregory; Langselius, Oliver; Thorne, Jennifer; Marconi, Vincent; Coburn, Sally; Lang, Raynell; Colasanti, Jonathan; Silverberg, Michael; Napravnik, Sonia; Loutfy, Mona; Karris, Maile; Sterling, Timothy; Burkholder, Greer; Althoff, Keri; Puhan, Milo

VERSION 1 - REVIEW

**REVIEWER NAME**	Kim, Jieun
**REVIEWER AFFILIATION**	Georgetown University School of Nursing & Health Studies
**REVIEWER CONFLICT OF INTEREST**	None
**AI DISCLOSURE**	No
**DATE REVIEW RETURNED**	06-Dec-2024

**GENERAL COMMENTS**	I am a medical student so I'm not sure if I am able to provide in depth comments about the technicality of the statin use such as dosing or intervals. However, I thought that this research was particularly impactful due to the 13.8% benefit harm analysis number being very quantitative. The data was presented very objectively in this manner, also when different percentages of risks were discussed (ex. diabetes vs myopathy), showing what side effect should be prioritized for physicians. I liked that although big picture trends in statin use and its benefits and side effects were presented at first, the paper went much further in depth in different types of statins and age brackets. I think that sex differences could be highlighted a bit more either in this paper or a future project, since there were higher percentages of men than women included in the study. This was understandable because it is an observational study and also due to the nature of HIV affecting more men than women in North America, but I wonder if there were important sex differences that were not discussed in the article, especially because sex differences do exist in CVD, diabetes, etc. Possibly, this article could be improved if there were differences in risk prevalences in males vs females, just as was done for the age bracket breakdown analysis. Overall, I thought that the results were impactful due to the 10 year longitudinal follow-up of CVD management and other cumulative risks. The limitations section showed many areas for future research which was exciting as well. Great population health study with great sample size and power. Thank you for allowing me to peer review this article, I hope I was at least a bit helpful.

**REVIEWER NAME**	McGettigan, Patricia

**REVIEWER AFFILIATION**	Queen Mary University of London
**REVIEWER CONFLICT OF INTEREST**	None
**AI DISCLOSURE**	No
**DATE REVIEW RETURNED**	09-Dec-2024

**GENERAL COMMENTS**	Review of ‘Statins for primary prevention of cardiovascular events in persons with HIV: A target trial and modelling study’<br><br>Overall<br>• Target trial emulation studies appear increasingly as access to large real world datasets and common data models grows, methodological skills develop, and guidance on their conduct, including from medicines regulators, evolves. Their potential to inform clinical practice, especially where RCTs are confined to limited populations and/or are unlikely to ever be conducted in broader / specific populations relevant for patients and clinical practitioners is great.<br>• This study addresses a clinically relevant area in an under-studied population in terms of CV risk.<br><br>Background<br>The question of applicability / generalisability of the findings of the REPRIEVE study are of particular interest to PWH and clinicians caring for them. REPRIEVE reported that CVD primary prevention with pitavstatin (v placebo) reduced the risk of major CV adverse events in people with HIV (PWH) who have with low-moderate CV risk. The caveats with generalising REPRIEVE to PWH broadly are multiple, not least that REPRIEVE is a single, non-replicated RCT using a single uncommonly-used statin, pitavstatin; though including 12 countries, over half of REPRIEVE’s 7769 patients were recruited in the US and Brazil, just 213 in Europe (Spain); it’s unclear if the effect is a pitavastatin-specific effect, a statin class effect, or more broadly, an effect of lipid lowering irrespective of therapy type.<br><br>In the UK, BHIVA issued a rapid recommendation that statin therapy should be offered as primary prevention to PWH aged 40y or over irrespective of lipids and baseline CV risk. For clinicians, whether they are HIV specialists or GPs who do the bulk of statin prescribing, this may be problematic because, for GPs especially, BHIVA advice does not align with guidance on offering statins to people without HIV. For PWH, BHIVA advice might be considered an unwelcome differentiation factor.<br>Realistically, it is unlikely that a RCT testing another statin / other statins / other LLDs will be conducted. This new study is therefore welcome. The authors conducted an open-label target trial emulation and modelling study using real world (observational) data from NA-ACCORD, US/Canada-based, with >20 cohorts of PHW, 85% of whom were male. The intention was to evaluate the effects of statins on primary CVD incidences and harm outcomes in PWH.<br><br>Methods<br>It is a bit difficult to follow the methods text, especially for readers not familiar with TTE. Potentially helpful information is relegated to the supplementary folder – Table S1 sets out the protocol components (sparsely), specifications and emulations in a Hernan & Roberts-style Framework. This is helpful and would be even more so for readers if it appeared in the main text.

	The target trial protocol was emulated using observational data from NA-ACCORD. There is little description of NA-ACCORD or its data. Supplementary documents include a list of the contributing cohorts; detail is absent, e.g., on data elements included in the datasets, record linkages, alignment of definitions and categorisations, degrees of data missingness, whether a common data model was used (NA_ACCORD cohorts supplementary information suggests this might be the case, 'Each contributing cohort (listed below) submits data annually in a standardized format to the Data Management Core…' but it is not clear.) Both Table S1 and text suggest to the reader that NA-ACCORD is a fairly homogeneous population. This is unlikely but there are no data to evaluate either way. Reference 14 describing NA-ACCORD is 17-years old – there needs to be clear and specific assurance regarding its current content, data-inclusion, quality and comprehensiveness, including linkages with prescription dispensing data and clinical records which likely have evolved in the interim. The population is dominantly male, greatly affecting generalisability of findings.<br><br>Table S1 is helpful but has omissions. There is scant detail on eligibility criteria other than having no prior CVD or statin initiation, specified lipid ranges, and a list of loosely defined contraindications, e.g., 'renal and liver problems' are contraindications, but are undefined; current use of erythromycin and clarithromycin are contraindications, presumably because they are CYP3A4 inhibitors, but what about other (potent) inhibitors, e.g., fluconazole, fluticasone? Of interest to the outcomes is how adherence was specified for statin initiators and for HIV therapy or how switching or non-adherence were considered.<br><br>Some omissions can be partly filled by reading the Methods text, e.g., dealing with missingness has a sentence of explanation; missing here appears to refer to missing values in collected data. Related here, the emulated randomisation adjusts for multiple potential baseline confounders (Table S1). This implies that all of these data were available from NA-ACCORD to predefined levels of acceptability. It's not explicit that this was the case.<br>The Discussion limitations text nods at this, noting that 'not all NA-ACCORD cohorts conduct outcome adjudication'. Other data missingness is not mentioned, e.g., data such as adherence, dose, switching, cessations, that would be meaningful for analyses but were not collected. Sensitivity analyses aimed to take account of some of these.<br>Table 1 provides data on years of enrolment in NA-ACCORD. In the 2000-2019 timespan covered, HIV therapy has changed considerably, esp in terms of exposure to therapies with greater / less inherent CV risk, even after 2006 when 72% were enrolled (Results p7/41). Statistical analyses text (p4/41) notes, without any detail, that year of enrolment was considered in the emulation matching to account for ARV and guideline changes over time. Related to this is the duration of HIV infection – Reference 1 notes HIV per se as a CV risk factor; it's not clear if duration of HIV infection was considered or if ARV commencement date was a proxy for duration. The Supplementary plots for covariate balance do not include these factors.<br><br>Baseline confounders that were adjusted-for in the emulated randomisation included diabetes (11% -12% of both initiators and non-initiators); AHA guidance is that diabetes per se is an indication for statin therapy; the rationale for inclusion is unclear given

	longstanding RCT evidence for benefit of statin primary prevention in people with diabetes. Given that diabetic patients were included, were initiator / non-initiator baseline difference in the duration / severity / control of diabetes considered too?<br>Initiation / non-initiation: “We defined treatment initiation as the first statin prescription and control (non-initiation) as the first development of a statin indication, including LDL ≥ 190 mg/dL, TC ≥ 290 mg/dL, a 10-year CVD risk of ≥7.5% for those with LDL of 70–189 mg/dL, or diabetic patients with LDL ≥70 mg/dL and having no contraindications.” Time-zero is different in initiators and non-initiators raising the possibility of biases, both selection and immortal time potentially, but this is not discussed.<br>The basis of the lipid parameters selected as indications for statin therapy is not referenced or discussed; similarly, the 10-year CV risk score where there is no information on its construction / algorithm or how it aligned with scores used elsewhere. Both, especially CV risk score subgroups are central to the interpretation of the findings. For non-US readers, the lipid measures need to be stated in mmol/L as well as mg/dL.<br><br>Sensitivity analyses were conducted for adherence (prescription duration >12-months), statin types, baseline age, CVD risk score, hypertension, InSTI use, diabetes and also included random placebo test.<br>Analyses are intention to treat; given that the data are observational, per protocol analyses are likely relevant too but are not discussed.<br>Cox proportional hazard models and use of the Kaplan-Meier estimator seem appropriate but should have methodology expert evaluation.<br><br>Benefit harm: Preferences from non-HIV populations in Switzerland and Ethiopia were used, adjusted for the current study but without clarity on adjustments.<br><br>Results<br>The results report that higher baseline CV risk aligned with greater 10-y CV benefit from statin therapy.<br>Main analyses are not distinguished from sensitivity analyses. CV 10-y risks were categorised as <7.5%, ≥7.5–10.0%, >10.0–15.0%, and ≥15.0%; they were included in the sensitivity analyses. Table 1 shows the median 10-y CV risk score, but it is not clear in the manuscript that CV risk categories were specified a priori; similarly for age categories also included in sensitivity analyses.<br>Assuming a priori CV 10-y risk stratification, the results highlight the undesirability of a headline single risk reduction measure, tempting as it is to report, and of losing nuances of the study that are of greatest decision-making assistance in patient-healthcare provider discussions about benefits / downsides of statin primary prevention.<br><br>Use duration / adherence proxy of >12-months prescriptions impacted benefit and harm – makes sense - but is hard to reconcile with 12-months statin use, which is short with CV impact, unless most with >12-months use used statins for considerably longer.<br>On the harm side, statin initiators had a small increase in diabetes risk.<br><br>The explorations of preferences and risk thresholds are extremely interesting but are buried in supplementary data and need better explanation for readers.

	Discussion<br>The discussion is balanced in many respects. The findings potentially refine evidence for primary prevention in PWH according to CV risk level. This is important given the harms associated with statin therapy, notably diabetes which of itself increases CV risk. https://www.sciencedirect.com/science/article/abs/pii/S0735109724097742?via%3Dihub<br>Nuances of the findings are discussed in Paragraph 2 of the Discussion. These are relevant for any application of the findings to clinical practice, especially the impact of co-morbidity and frailty. They also underline the need to be clear CV risk strata were pre-planned.<br><br>The impact of patient preference on net benefit of statins is intuitively 'true' for readers who care for PWH, reflecting real-life clinic discussions / experiences. It deserves further exploration in future, especially as real world data sets become capable of including preference and patient-reported outcomes information and systematically evaluating associated outcomes.<br><br>The TTE findings align overall with REPRIEVE but are more nuanced and could potentially improve blanket recommendations on primary prevention with statins for PWH that were issued by some HIV specialist groups following REPRIEVE publication. They also highlight one of REPRIEVE's multiple limitations in using a statin uncommonly prescribed in clinical practice.<br><br>Some study limitations are well addressed. Others, especially those affecting generalisability to PWH populations outside of North America and who are not dominantly male need discussion. Limitations / benefits of TTE also deserve some discussion. The reference describing NA-ACCORD is very dated but no information is provided on the current state of the dataset or the contributing cohorts – this is crucial for insight on the reliability and generalisability of the current study. NA-ACCORD data completeness, gaps / deficits, quality assurance measures are not explicit – there is little information and readers are provided no insight on the quality of the data.<br>See also earlier notes on the need for explanation on lipid parameters and 10-year CV risk categories.<br>Conclusions are reasonable.

**REVIEWER NAME**	Ponatshego, Ponego
**REVIEWER AFFILIATION**	Botswana-Harvard AIDS Institute Partnership, Clinical Servises
**REVIEWER CONFLICT OF INTEREST**	None
**AI DISCLOSURE**	No
**DATE REVIEW RETURNED**	07-Jan-2025

**GENERAL COMMENTS**	An overall well designed study and a well written manuscript, with the potential to contribute significant knowledge to the important field of aging with HIV. As a Sub Saharan based clinician, aging with HIV is of special interest as there is a significantly large such a population.

**REVIEWER NAME**	Davies, Neil
**REVIEWER AFFILIATION**	UCL, Division of Psychiatry and Department of Statistical Science

**REVIEWER CONFLICT OF INTEREST**	None
**AI DISCLOSURE**	No
**DATE REVIEW RETURNED**	08-Jan-2025

**GENERAL COMMENTS**	bmjmed-2024-001132 Statins for primary prevention of cardiovascular events in<br>persons with HIV: A target trial and modelling study<br><br>This paper estimates the effectiveness and benefit harm of different statins for primary prevention of CVD for people with HIV. They use a cohort of people enrolled in a study of AIDS (NA-ACCORD), and a target trial approach.<br><br>This is an interesting paper that has been well conducted. There are a few areas that could be improved. The description of causation could be refined, and the focus on personalization does not entirely convince me. I noted some areas in the paper that could be strengthened or clarified. I hope these comments are helpful.<br><br>Abstract<br>It would be helpful to downplay the lack of statistical significance for cognitive impairment, myopathy and rhabdomyolysis, and rather interpret the upper and lower confidence intervals as indications of the size of association that is unlikely given your results.<br><br>Conclusions - tailoring statin treatment to individual risks – your analyse don't address this hypotheses (is personalisation likely to be important or effective?) rather average effect of treatment across the population. You could consider rephrasing this to either justify why your results support personalisation, or dropping this sentence, as it is not really the focus of your study.<br><br>P5L57 – Why did you restrict to individuals with low risk of CVD and low levels of LDLc?<br>P6L15 – How many patients in your data received lovastatin and simvastatin?<br>P8L23 – how do these relative differences in effectiveness compare to published network meta-analyses in the general population? For the discussion if not already there.<br>P8L25 – Could you confirm re the lower risk of diabetes with pravastatin? Is this an odds-ratio or risk difference? Similarly with the other classes of statins, would be worth confirming what the parameter is here.<br>P8L37 "The benefit-harm balance showed only moderate variation between demographic and clinical subgroups" Could you confirm –your conclusions in your abstract suggested personalisation was potentially important given your results?<br>P10L25 "The findings demonstrated that statin treatment reduced any CVD events by 21% but increased events of diabetes by 12%." This would perhaps be better phrased as "Patients who initiated statin treatment had 21% lower rates of CVD events, but 12% higher rates of diabetes. I am very much on board with describing observational studies clearly as estimates of a causal effect, but here I think it's probably clearer to your reader to simply state what you found, rather than putting too much weight on the causal interpretation, which as I'm sure you'll get to, requires all the usual strong assumptions.

	P10L32 – “risk thresholds differed” where are the statin results? The risk thresholds in Table S8 don’t have CI, so it’s not possible to determine if these differences are meaningful. It would be useful to add CI to this table, and check whether these differences are more than we would expect by chance.<br>P10L37 “demonstrate potential interactions of statin effects by subgroup characteristics that could add to the subgroup variation. But these effects should be interpreted with caution, as the target trial design and analysis may not be sufficiently robust for subgroup estimation.” Which results are you referring to here? Is the issue that the TT approach is not sufficiently robust (bias), or that your subgroup analyses are likely to be underpowered (precision)?<br><br>P10L41 “finding of our study was that the net benefit of statins was sensitive to patient preferences, implying<br>that use of one-size-fits-all thresholds could be overly simplistic. Treatment initiations should be personalised to<br>individual patients factoring in their outcome risks and preferences.” This is a quite a strong claim based on the data you are presenting. Surely we would need an RCT of such personalisation to demonstrate personalisation of this sort would improve patient outcomes?<br><br>P10L48 – Would be worth plotting your results versus previously published results (e.g. in a coefficient plot). “might explain the difference…” it would be far easier for your readers if you presented this difference in a plot. Your explanation here is compelling, and it’s ok and informative if your results differ from previously published RCTs.<br><br>P11L11 – “although we endeavoured to control for such variance.” How did you control for this variance?<br><br>P11L29 – Here your models assumed the effect of preference. I do not think this study demonstrates that patients outcomes will be improved if patient treatment is personalised to people’s preferences about the relative importance of CVD versus diabetes. This is an interesting question, but it is not easily answerable from the data you have, nor (in my view), do you need to answer it – your data and results are more than interesting enough.<br><br>Limitations – the big limitation to the study is residual confounding. If patients who are healthier or more health conscious ask for and initiate statin treatment, then you may overestimate the effect of statins on each of your outcomes. Measurement error on the outcomes, exposure and covariates. Missing not entirely at random variables.<br><br>Please could you upload the code used to clean and analyse your data, and either add the pre-registration/protocol, or add a statement that the analysis wasn’t pre-registered? Please could you also add any study approval number for your access to NA-ACCORD data.

VERSION 1 – AUTHOR RESPONSE

	Reviewer's comments	Author's reply
**Reviewer 1**	**Miss Jieun Kim, Georgetown University School of Nursing & Health Studies, National Institute for Medical Research Mwanza Research Centre.**	
	I am a medical student so I'm not sure if I am able to provide in depth comments about the technicality of the statin use such as dosing or intervals. However, I thought that this research was particularly impactful due to the 13.8% benefit harm analysis number being very quantitative. The data was presented very objectively in this manner, also when different percentages of risks were discussed (ex. diabetes vs myopathy), showing what side effect should be prioritized for physicians. I liked that although big picture trends in statin use and its benefits and side effects were presented at first, the paper went much further in depth in different types of statins and age brackets. I think that sex differences could be highlighted a bit more either in this paper or a future project, since there were higher percentages of men than women included in the study. This was understandable because it is an observational study and also due to the nature of HIV affecting more men than women in North America, but I wonder if there were important sex differences that were not discussed in the article, especially because sex differences do exist in CVD, diabetes, etc. Possibly, this article could be improved if there were differences in risk prevalences in males vs females, just as was done for the age bracket breakdown analysis. Overall, I thought that the results were impactful due to the 10-year longitudinal follow-up of CVD management and other cumulative risks. The limitations section showed many areas for future research which was exciting as well. Great population health study with great sample size and power. Thank you for allowing me to peer review this article, I hope I was at least a bit helpful.	Thank you for highlighting the importance of this topic. We also share the concern about the disproportionate sample size between men and women in the NA-ACCORD cohorts. As stated in the paper, this limitation can be addressed by utilizing other datasets outside the US, where sufficient sample for women could be available.<br><br>While the only trial (REPRIEVE) showed no difference in effect between men and women, the findings have not yet been replicated. The trial also tested a single uncommonly used statin.
Reviewer 2	Prof. Patricia McGettigan, Queen Mary University of London<br>Comments to the Author	
		We greatly appreciate the reviewer's insightful and valuable comments, most of which support our findings. We provide our reflections or replies where applicable.

**1**	Review of ‘Statins for primary prevention of cardiovascular events in persons with HIV: A target trial and modelling study’<br><br>Overall<br>• Target trial emulation studies appear increasingly as access to large real world datasets and common data models grows, methodological skills develop, and guidance on their conduct, including from medicines regulators, evolves. Their potential to inform clinical practice, especially where RCTs are confined to limited populations and/or are unlikely to ever be conducted in broader / specific populations relevant for patients and clinical practitioners is great.<br>• This study addresses a clinically relevant area in an under-studied population in terms of CV risk.<br><br>Background<br>The question of applicability / generalisability of the findings of the REPRIEVE study are of particular interest to PWH and clinicians caring for them. REPRIEVE reported that CVD primary prevention with pitavstatin (v placebo) reduced the risk of major CV adverse events in people with HIV (PWH) who have with low-moderate CV risk. The caveats with generalising REPRIEVE to PWH broadly are multiple, not least that REPRIEVE is a single, non-replicated RCT using a single uncommonly-used statin, pitavstatin; though including 12 countries, over half of REPRIEVE’s 7769 patients were recruited in the US and Brazil, just 213 in Europe (Spain); it’s unclear if the effect is a pitavastatin-specific effect, a statin class effect, or more broadly, an effect of lipid lowering irrespective of therapy type.<br><br>In the UK, BHIVA issued a rapid recommendation that statin therapy should be offered as primary prevention to PWH aged 40y or over irrespective of lipids and baseline CV risk. For clinicians, whether they are HIV specialists or GPs who do the bulk of statin prescribing, this may be problematic because, for GPs especially, BHIVA advice does not align with guidance on offering statins to people without HIV. For PWH, BHIVA advice might be considered an unwelcome differentiation factor. Realistically, it is unlikely that a RCT testing another statin / other statins / other LLDs will be conducted. This new study is therefore welcome. The authors conducted an open-label target trial emulation and modelling study using real world (observational) data from NA-ACCORD, US/Canada-based, with >20 cohorts of PHW, 85% of whom were male. The intention was to evaluate the	We agree with the reviewer’s reflection on the study’s importance and its added value beyond the REPRIEVE trial.

	effects of statins on primary CVD incidences and harm outcomes in PWH.	
2	Methods<br>It is a bit difficult to follow the methods text, especially for readers not familiar with TTE. Potentially helpful information is relegated to the supplementary folder – Table S1 sets out the protocol components (sparsely), specifications and emulations in a Hernan & Roberts-style Framework. This is helpful and would be even more so for readers if it appeared in the main text.	Thank you have moved it back to the main text as Table 1. We initially moved Table S1 to reduce the number of tables in the main text. However, we agree that including it in the main text would better guide readers through the paper.
3	The target trial protocol was emulated using observational data from NA-ACCORD. There is little description of NA-ACCORD or its data. Supplementary documents include a list of the contributing cohorts; detail is absent, e.g., on data elements included in the datasets, record linkages, alignment of definitions and categorisations, degrees of data missingness, whether a common data model was used (NA_ACCORD cohorts supplementary information suggests this might be the case, 'Each contributing cohort (listed below) submits data annually in a standardized format to the Data Management Core…' but it is not clear.) Both Table S1 and text suggest to the reader that NA-ACCORD is a fairly homogeneous population. This is unlikely but there are no data to evaluate either way. Reference 14 describing NA-ACCORD is 17-years old – there needs to be clear and specific assurance regarding its current content, data-inclusion, quality and comprehensiveness, including linkages with prescription dispensing data and clinical records which likely have evolved in the interim. The population is dominantly male, greatly affecting generalisability of findings.	Please see Table 1 in the original manuscript (or Table 2 in the revised) and Table S3 in the supplementary materials, which outline the NA-ACCORD's important variables with their distributions. Relevant variables are also listed in the text, particularly in the sections on eligibility and analysis.<br><br>Regarding references about quality of the cohorts, we followed the NA-ACCORD Steering Committee's guidance to cite the recommended paper.
4	Table S1 is helpful but has omissions. There is scant detail on eligibility criteria other than having no prior CVD or statin initiation, specified lipid ranges, and a list of loosely defined contraindications, e.g., 'renal and liver problems' are contraindications, but are undefined; current use of erythromycin and clarithromycin are contraindications, presumably because they are CYP3A4 inhibitors, but what about other (potent) inhibitors, e.g., fluconazole, fluticasone? Of interest to the outcomes is how adherence was specified for statin initiators and for HIV therapy or how switching or non-adherence were considered.<br><br>Some omissions can be partly filled by reading the Methods text, e.g., dealing with missingness has a sentence of explanation; missing here appears to refer to missing values in collected data. Related here, the emulated randomisation adjusts for multiple potential	We provided the detailed eligibility criteria, including no exposure to statins, no history of CVD as we focus on primary CVD prevention, eligibility to statins, including age, stratification by CVD risk scores, lipid levels, diabetes status, as well as several contraindications. We believe these are the main eligibility attributes. However, we missed to mention all drug inhibitors, specifically fluconazole and fluticasone. We accounted these in the analyses thanks to the NA-ACCORD's comprehensive data on medications. We have mentioned them now.<br><br>The reviewer is correct that we adjusted for all relevant baseline confounding factors. Due to space constraints (as our study addresses causal estimations for multiple outcomes and includes a benefit-harm analysis), we summarized key methods (e.g.,

	baseline confounders (Table S1). This implies that all of these data were available from NA-ACCORD to predefined levels of acceptability. It's not explicit that this was the case.	multiple imputations) in the main text and provided full details and diagnostic plots in the supplementary materials.<br><br>For adherence: We understand the reviewer's concern. However, the focus of the paper is on the intention-to-treat (ITT) effect contrast. While we initially analyzed per -protocol effects using sequential target trial emulations and pooled logistic regression accounting for censoring/adherence, high levels of missingness in critical time-varying covariates (e.g., lipids, BMI) rendered imputation and estimation unreliable. However, we performed some sensitivity analyses, as detailed in the paper, including limiting data to long-term users as well as excluding those with >2-month discontinuations.
5	The Discussion limitations text nods at this, noting that 'not all NA-ACCORD cohorts conduct outcome adjudication'. Other data missingness is not mentioned, e.g., data such as adherence, dose, switching, cessations, that would be meaningful for analyses but were not collected. Sensitivity analyses aimed to take account of some of these.	Thank you for the feedback. These points are addressed in the limitations section of the discussion, along with outcome adjudication. Please see the following excerpt from the discussion:<br><br>*"Our study provided a nuanced analysis of the effectiveness and safety of statins using a target trial, as well as risk thresholds to guide decision-making for statin treatment. However, the limitations should also be accounted for when interpreting the findings. We did not perform dose-effect analyses due to limited dose data. Future research should address the underrepresentation of women in the cohorts. Our assumption of adherence to statins may be overstated. It is possible that there was significant non-adherence due to harm outcomes, polypharmacy, drug interactions, and other reasons. In a controlled study where adherence is measured and adjusted for, the effect of statin could be greater than what we found, as clued by our explorative analyses limited to long-term statin use. This should be addressed in other data with adherence indicator. We mainly estimated intention-to-treat causal effects, and there might be differential switching of treatment assignments as in RCTs due to changes in patient prognosis after enrolment in the target trial………"*
6	Table 1 provides data on years of enrolment in NA-ACCORD. In the 2000-2019 timespan covered, HIV therapy has changed considerably, esp in terms of exposure to therapies with greater / less inherent CV risk, even after 2006 when 72% were enrolled (Results p7/41). Statistical analyses text (p4/41) notes, without any detail, that year of enrolment was considered in the	Both year of enrollment and duration of HIV infection (represented by ARV commencement as proxy) were critically controlled in the analysis. Patients were matched within the same year of enrollment, ensuring no initiator was matched with a control from a different year. This is reflected in the text and in the diagnostic plots in the

	emulation matching to account for ARV and guideline changes over time. Related to this is the duration of HIV infection – Reference 1 notes HIV per se as a CV risk factor; it's not clear if duration of HIV infection was considered or if ARV commencement date was a proxy for duration. The Supplementary plots for covariate balance do not include these factors.	supplementary materials (labeled as 'Year of entry').<br><br>However, since ARV commencement is similar to enrollment to ART in most cases, both variables had overlapping information. To void propensity score instability, we took 'year of enrolment as the main predictor'. More importantly, the control of HIV was accounted for through variables such as CD4 count, viral load, and treatment status.
7	Baseline confounders that were adjusted-for in the emulated randomisation included diabetes (11% -12% of both initiators and non-initiators); AHA guidance is that diabetes per se is an indication for statin therapy; the rationale for inclusion is unclear given longstanding RCT evidence for benefit of statin primary prevention in people with diabetes. Given that diabetic patients were included, were initiator / non-initiator baseline difference in the duration / severity / control of diabetes considered too?<br>Initiation / non-initiation: "We defined treatment initiation as the first statin prescription and control (non-initiation) as the first development of a statin indication, including LDL ≥ 190 mg/dL, TC ≥ 290 mg/dL, a 10-year CVD risk of ≥7.5% for those with LDL of 70–189 mg/dL, or diabetic patients with LDL ≥70 mg/dL and having no contraindications." Time-zero is different in initiators and non-initiators raising the possibility of biases, both selection and immortal time potentially, but this is not discussed.	Statins are generally recommended for individuals with diabetes when their LDL exceeds 70 mg/dL, as it is a strong CVD predictor. However, statins also increase diabetes risk, as shown in both HIV and non-HIV populations, including our study. It remains uncertain whether the benefits of statins in reducing diabetes-related CVD risk outweigh their risks of elevated blood sugar.<br><br>Diabetes control was accounted for by adjusting for diabetes medication use, but blood sugar levels at time zero were unavailable (only at the time of diagnosis was available which did not align with the time-zero for the target trial).<br><br>The reviewer has rightly noted concerns about time zero. However, time zero was carefully aligned for both groups, a critical step to reduce selection and immortal time bias— very important issues than confounding Time zero was defined as the first statin initiation for users and the time they became eligible to initiate statins (but did not initiate) for non-users.<br><br>For better readability, we added the term 'time zero' in the revised manuscript, such as *'We defined **time zero** as treatment initiation…'.*
8	The basis of the lipid parameters selected as indications for statin therapy is not referenced or discussed; similarly, the 10-year CV risk score where there is no information on its construction / algorithm or how it aligned with scores used elsewhere. Both, especially CV risk score subgroups are central to the interpretation of the findings.	The CVD estimator, the ACC/AHA Pooled Cohort Equations, was cited. However, the REPRIEVE trial protocol, a reference for the CVD risk stratification, was previously not cited but has now been added to the citation.
9	Sensitivity analyses were conducted for adherence (prescription duration >12-months), statin types, baseline age, CVD risk score, hypertension, InSTI use, diabetes and also included random placebo test. Analyses are intention to treat; given that the data are observational, per protocol analyses are likely relevant too but are not discussed. Cox proportional hazard	Please see reply to comment #4 above regarding adherence and per-protocol analysis.<br><br>We agree with the reviewer that Cox Proportional Hazard works well for intention-to-treat analysis and that is what we did.

	models and use of the Kaplan-Meier estimator seem appropriate but should have methodology expert evaluation.	
10	Results<br>The results report that higher baseline CV risk aligned with greater 10-y CV benefit from statin therapy.<br>Main analyses are not distinguished from sensitivity analyses. CV 10-y risks were categorised as <7.5%, ≥7.5–10.0%, >10.0–15.0%, and ≥15.0%; they were included in the sensitivity analyses. Table 1 shows the median 10-y CV risk score, but it is not clear in the manuscript that CV risk categories were specified a priori.<br>Assuming a priori CV 10-y risk stratification, the results highlight the undesirability of a headline single risk reduction measure, tempting as it is to report, and of losing nuances of the study that are of greatest decision-making assistance in patient-healthcare provider discussions about benefits / downsides of statin primary prevention.	We have indicated now that the subgroups were defined a priori in the text.<br><br>We completely agree on the importance of emphasizing subgroups to provide valuable insights. However, we also want to exercise caution regarding overinterpretation of subgroup causal effects. This is because the conditional exchangeability assumption is challenging to satisfy at the subgroup level, and some effects may not be consistent across subgroups. Nonetheless, the subgroups effects should serve as initial evidence to replicate in other studies.<br><br>*We discussed this in the discussion as "While the reported risk threshold variation stemmed from differences in outcome risk of harms, we also attempted to demonstrate potential interactions of statin effects by subgroup characteristics that could add to the subgroup variation (Table S4)."*
11	Use duration / adherence proxy of >12-months prescriptions impacted benefit and harm – makes sense - but is hard to reconcile with 12-months statin use, which is short with CV impact, unless most with >12-months use used statins for considerably longer.<br>On the harm side, statin initiators had a small increase in diabetes risk.	We agree that the minimum duration ~12 months of statin use could be short to observe CVD events. However, this analysis should serve as explorative analysis to assess adherence influence on the statin effect. In fact, >12 months contain always years of statin use.<br><br>Limiting the analysis to individuals with much longer statin use significantly reduced the sample size, which limited statistical power.
12	The explorations of preferences and risk thresholds are extremely interesting but are buried in supplementary data and need better explanation for readers.	Thank you for highlighting the importance of preferences, which are proxies for patient treatment goals and willingness to accept harms for benefits. We agree that these are important for refining decision-making. We moved the plots to supplementary mainly due to space constraints, but the main results are in the main text. We have also added the results in the abstract (please see the revised abstract).
13	Discussion<br>The discussion is balanced in many respects. The findings potentially refine evidence for primary prevention in PWH according to CV risk level. This is important given the harms associated with statin therapy, notably diabetes which of itself increases CV risk. https://www.sciencedirect.com/science/article/abs/pii/S0735109724097742?via%3Dihub<br>Nuances of the findings are discussed in Paragraph 2 of the Discussion. These are relevant for any application of	Thank you for your valuable feedback; we agree with your comments.<br><br>Regarding references about quality of the cohorts, we followed the NA-ACCORD Steering Committee's guidance to cite the recommended paper.<br><br>Please also refer to the NA-ACCORD page (https://naaccord.org/available-data-elements?utm_source=chatgpt.com) about important attributes collected. We have

	the findings to clinical practice, especially the impact of co-morbidity and frailty. They also underline the need to be clear CV risk strata were pre-planned.<br><br>The impact of patient preference on net benefit of statins is intuitively 'true' for readers who care for PWH, reflecting real-life clinic discussions / experiences. It deserves further exploration in future, especially as real world data sets become capable of including preference and patient-reported outcomes information and systematically evaluating associated outcomes.<br><br>The TTE findings align overall with REPRIEVE but are more nuanced and could potentially improve blanket recommendations on primary prevention with statins for PWH that were issued by some HIV specialist groups following REPRIEVE publication. They also highlight one of REPRIEVE's multiple limitations in using a statin uncommonly prescribed in clinical practice.<br><br>Some study limitations are well addressed. Others, especially those affecting generalisability to PWH populations outside of North America and who are not dominantly male need discussion. Limitations / benefits of TTE also deserve some discussion. The reference describing NA-ACCORD is very dated but no information is provided on the current state of the dataset or the contributing cohorts – this is crucial for insight on the reliability and generalisability of the current study. NA-ACCORD data completeness, gaps / deficits, quality assurance measures are not explicit – there is little information and readers are provided no insight on the quality of the data.<br>See also earlier notes on the need for explanation on lipid parameters and 10-year CV risk categories.<br>Conclusions are reasonable.	included the most important variables in the analysis, as shown in Table 2 (matched sample) and Supplementary Table 3 (unmatched sample with a larger size).<br><br>We greatly appreciate the reviewer's in-depth insights and valuable comments on the paper.
**Reviewer 3**	**Dr. Ponego Ponatshego, Botswana-Harvard AIDS Institute Partnership**	
	Comments to the Author<br>An overall well-designed study and a well written manuscript, with the potential to contribute significant knowledge to the important field of aging with HIV. As a Sub-Saharan based clinician, aging with HIV is of special interest as there is a significantly large such a population.	Thank you for your feedback and for highlighting the importance of this topic. We agree that CVD is a significant health issue in the HIV population because of the elevated risk due to ARTs and chronic inflammation, as well as aging.

**Reviewer 4**	**Dr. Neil Davies, University of Bristol**	
1	bmjmed-2024-001132 Statins for primary prevention of cardiovascular events in<br>persons with HIV: A target trial and modelling study<br><br>This paper estimates the effectiveness and benefit harm of different statins for primary prevention of CVD for people with HIV. They use a cohort of people enrolled in a study of AIDS (NA-ACCORD), and a target trial approach.<br><br>This is an interesting paper that has been well conducted. There are a few areas that could be improved. The description of causation could be refined, and the focus on personalization does not entirely convince me. I noted some areas in the paper that could be strengthened or clarified. I hope these comments are helpful.	Thank you for your insightful comments and constructive feedback. We appreciate your suggestions and address each point in detail below.
2	Abstract<br>It would be helpful to downplay the lack of statistical significance for cognitive impairment, myopathy and rhabdomyolysis, and rather interpret the upper and lower confidence intervals as indications of the size of association that is unlikely given your results.	We agree with the reviewer, but we believe it is important not to overlook the harm, regardless of reaching statistical significance, as studies in non-HIV populations have highlighted this harm. To strike a balance, we reported the finding while clearly noting its lack of statistical significance, ensuring transparency in presenting all relevant results.
3	Conclusions - tailoring statin treatment to individual risks – your analyse don't address this hypotheses (is personalisation likely to be important or effective?) rather average effect of treatment across the population. You could consider rephrasing this to either justify why your results support personalisation, or dropping this sentence, as it is not really the focus of your study.	Thank you for the great observation. In the abstract, we initially reported the CVD thresholds by subgroups and patient preferences, with detailed results provided in the main text. However, we have now incorporated specific thresholds for these variations from the main body of the text to provide more detailed insights at personal level. We clarify that personalisation in our study refers to risk thresholds or decision-making regarding statin initiation, not to individualized causal treatment effects (ITE) of statins.<br><br>Risk thresholds were not determined based only on treatment effects, but we also considered CVD risks (calculated from personal risk factors) and patient preferences, both personal data resulting in tailored decision-making. We have reworded the term to "tailored" or "stratified decisions" where appropriate to better reflect this focus.

4	P5L57 – Why did you restrict to individuals with low risk of CVD and low levels of LDLc?	Firstly, we did not restrict the analysis to individuals with low LDL in the target trial. In the target trial, we included participants in the target trial those who initiated statins as well as controls who became eligible (e.g., LDL ≥190) but did not initiate statins.<br><br>Now, the importance of restricting LDL <190 (or similar criteria) was to ensure that exposure (statin initiation or indication development in the control) is clearly defined and occurs after study entry. This essential for causal inference to define time zero and avoid immortal time bias and selection bias by excluding periods where participants are ineligible for statins. It helps to clearly define the time zero (to initiate statins or to become eligible for statins) and allows a valid counterfactual framework of estimation and interpretation of the statin effects on CVD outcomes.
5	P6L15 – How many patients in your data received lovastatin and simvastatin?	The prescription rates in the NA-ACCORD cohort were 1.5% for lovastatin and 18.09% for simvastatin, respectively (see Table S9). However, as explained in the paper, these were excluded from the analysis due to their notable adverse effects or interactions.
6	P8L23 – how do these relative differences in effectiveness compare to published network meta-analyses in the general population? For the discussion if not already there.	The findings were quite similar to those in the general population, as compared in the discussion. For example, the HR for CVD outcomes in our study was 0.79, whereas meta-analyses in non-HIV populations report values across meta-analysis of different studies, with RR/HR 0.72 (95% CI: 0.64–0.81) to 0.77 (95% CI: 0.68–0.86). Similarly, the effect on type 2 diabetes in our study was 1.12 (95% CI: 1.01–1.25), compared to 1.09 (95% CI: 1.02–1.16) in non -HIV populations.
7	P8L25 – Could you confirm re the lower risk of diabetes with pravastatin? Is this an odds-ratio or risk difference? Similarly with the other classes of statins, would be worth confirming what the parameter is here.	Yes, it is HR and we have added it with each estimate.
8	P8L37 “The benefit-harm balance showed only moderate variation between demographic and clinical subgroups” Could you confirm –your conclusions in your abstract suggested personalisation was potentially important given your results?	Thank you for pointing this out. We have rephrased the term ‘moderate variation’ to avoid ambiguity or subjectivity. But we also added the main quantitative results by subgroup and preferences in the abstract. The subgroup results were all only in main text and supplementary in the original submission.<br><br>We agree that these variations support the personalized decision or tailored decisions.

9	P10L25 “The findings demonstrated that statin treatment reduced any CVD events by 21% but increased events of diabetes by 12%.” This would perhaps be better phrased as “Patients who initiated statin treatment had 21% lower rates of CVD events, but 12% higher rates of diabetes. I am very much on board with describing observational studies clearly as estimates of a causal effect, but here I think it’s probably clearer to your reader to simply state what you found, rather than putting too much weight on the causal interpretation, which as I’m sure you’ll get to, requires all the usual strong assumptions.	We agree and have made the adjustment. Thank you for the suggested rephrasing.
10	P10L32 – “risk thresholds differed” where are the statin results? The risk thresholds in Table S8 don’t have CI, so it’s not possible to determine if these differences are meaningful. It would be useful to add CI to this table, and check whether these differences are more than we would expect by chance.	The risk thresholds were estimated from a probability measure (see the figure). The probability itself, similar to a p-value for example, does not have a CI as it inherently represents a measure of uncertainty. However, the probability of net benefit was calculated by incorporating the estimates and uncertainties around the baseline risks, treatment effects, and preferences, as explained in the paper.<br>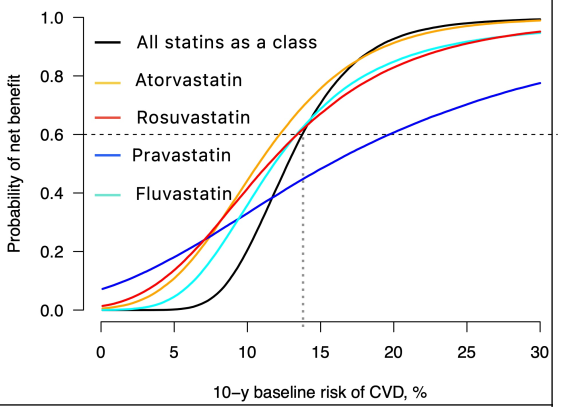
11	P10L37 “demonstrate potential interactions of statin effects by subgroup characteristics that could add to the subgroup variation. But these effects should be interpreted with caution, as the target trial design and analysis may not be sufficiently robust for subgroup estimation.” Which results are you referring to here? Is the issue that the TT approach is not sufficiently robust (bias), or that your subgroup analyses are likely to be underpowered (precision)?	Thank you for pointing this out. The results refer to the subgroup causal estimates, which can be found in Table S4 (now indicated in the text).<br><br>As the reviewer rightly commented, the subgroup causal estimates should not be overinterpreted because the conditional exchangeability assumption is challenging to satisfy at the subgroup level, and some effects may not be consistent across subgroups.
12	P10L41 “finding of our study was that the net benefit of statins was sensitive to patient preferences, implying that use of one-size-fits-all thresholds could be overly simplistic. Treatment initiations should be personalised to individual patients factoring in their outcome risks and preferences.” This is a quite a strong claim based on the data you are presenting. Surely we would need an RCT	Please refer to our response to **comment #3** regarding personalization. As discussed there, personalization in our study is not solely about causal ITE but about decision-making, encompassing all relevant parameters essential, including personalized outcome risks and patient preferences.

	of such personalisation to demonstrate personalisation of this sort would improve patient outcomes?	Moreover, while an RCT could provide additional insights, it is not strictly necessary. Patient preferences (*reflecting individual treatment goals and willingness to accept harms for benefits*) can be collected through surveys, such as DCE, BWS, time (person)-trade off…
12	P10L48 – Would be worth plotting your results versus previously published results (e.g. in a coefficient plot). "might explain the difference…" it would be far easier for your readers if you presented this difference in a plot. Your explanation here is compelling, and it's ok and informative if your results differ from previously published RCTs.	This is a great suggestion. However, given the extensive content in the main text, including details on causal estimates for multiple outcome and benefit-harm analyses, we opted to relegate even detailed results pertinent to our analysis to the supplementary materials to adhere to the word limit. We appreciate the suggestion.
13	P11L11 – "although we endeavoured to control for such variance." How did you control for this variance?	The word 'variance' may not be ideal, as it is usually associated with a statistical concept. We have replaced it with '*changes*.'<br><br>If the question relates to how we controlled for treatment changes over time, please refer to our response to **Reviewer 2**, **comment #6**, for further details.
14	P11L29 – Here your models assumed the effect of preference. I do not think this study demonstrates that patients outcomes will be improved if patient treatment is personalised to people's preferences about the relative importance of CVD versus diabetes. This is an interesting question, but it is not easily answerable from the data you have, nor (in my view), do you need to answer it – your data and results are more than interesting enough.	No, the study does not demonstrate that preference affects the causal effect of treatment (e.g., the clinical efficacy of statins). Instead, it shows that preferences influence the perceived net clinical benefit or quality of life by aligning outcomes with individual patient values.<br><br>While this approach does not directly measure clinical outcome improvements, it demonstrates how preferences could be integrated into decision-making to enhance patient-centered care.<br><br>This is analogous to incorporating utility measures, which are widely used to assess quality of life in health economic evaluations.
15	Limitations – the big limitation to the study is residual confounding. If patients who are healthier or more health conscious ask for and initiate statin treatment, then you may overestimate the effect of statins on each of your outcomes. Measurement error on the outcomes, exposure and covariates.	We agree with the reviewer that the presence of residual confounding and measurement error are generally difficult to be fully ruled out in real-world data, although we attempted to address these through tests such as placebo treatments. Another way, we tried to address this issue is adding proxy variables. For example, potential residual confounders could include physical exercise and diet, which are not collected in the NA-ACCORD dataset. However, we included most accessible confounding factors that could be proxies—for instance, BMI and lipids may provide indirect information for physical activity and diet. However, we generally share the reviewer's concern.

16	Please could you upload the code used to clean and analyse your data, and either add the pre-registration/protocol, or add a statement that the analysis wasn't pre-registered? Please could you also add any study approval number for your access to NA-ACCORD data.	The study was pre-registered within the NA-ACCORD system, and data access was secured through a formal application and agreement. Additionally, the majority of co-authors are investigators in the different NA-ACCORD cohorts.<br><br>While we are happy to share the code with interested individuals, we want to ensure it is clear and usable for external users. Please feel free to reach out to us directly for access.

VERSION 2 – REVIEW

**REVIEWER NAME**	McGettigan, Patricia
**REVIEWER AFFILIATION**	Queen Mary University of London
**REVIEWER CONFLICT OF INTEREST**	None
**AI DISCLOSURE**	No
**DATE REVIEW RETURNED**	20-Feb-2025

**GENERAL COMMENTS**	Thank you for addressing concerns and answering questions raised in the reviews of the manuscript.<br>Response issues:<br>1. Suggest remove fluticasone from the sentence "……and current use of antimicrobials (such as erythromycin, clarithromycin, fluconazole, and fluticasone) all assessed at the time of entry to the follow up" and simply stick to anti-microbials. (fluticasone is not an anti-microbial; levels rise excessively if used concomitantly with some HIV drugs such as atazanavir, lopinavir, ritonavir).<br>2. Decide between PWH (text) and PLWH (What is already known box)<br>The US measure of mg/dL is used throughout; simultaneous mmol/L equivalents for 3. 3. The US measure of mg/dL is used throughout; simultaneous mmol/L equivalents for UK-based BMJ Medicine??<br>4. The authors agree that the possibility of residual confounding is a limitation of the study. But it is not acknowledged in the discussion; it would seem wise to do so given the nature of the cohort, its time span, and the therapeutic evolution/s, both CV and HIV, that have occurred over the period.<br><br>This is an informative study - it will be welcomed by the HIV community of patients and their health carers for improving insights on holistic use of statins.

**REVIEWER NAME**	Davies, Neil
**REVIEWER AFFILIATION**	UCL, Division of Psychiatry and Department of Statistical Science
**REVIEWER CONFLICT OF INTEREST**	None
**AI DISCLOSURE**	No
**DATE REVIEW RETURNED**	07-Mar-2025

**GENERAL COMMENTS**	Thank you for considering my comments.

	Re pre-registration and analytic code - it would substantially strengthen the paper, and be useful for your readers to publish your protocol and code used to conduct this study. This is increasingly seen as a key part of reporting a study like this. You've done a great job on this analysis, and publishing the protocol and code would demonstrate this. However, I leave it to your and the editors' discretion.

VERSION 2 – AUTHOR RESPONSE

Reviewer #2: Prof. Patricia McGettigan, Queen Mary University of London

1) Suggest remove fluticasone from the sentence "……and current use of antimicrobials (such as erythromycin, clarithromycin, fluconazole, and fluticasone) all assessed at the time of entry to the follow up" and simply stick to anti-microbials. (fluticasone is not an anti-microbial; levels rise excessively if used concomitantly with some HIV drugs such as atazanavir, lopinavir, ritonavir).

Reply: Thank you very much for the careful observation. We adjusted it to be "….current use of medications with potential interactions with statins…" (Page4, Line 9). The list of medications are in the target trial protocol, table 1.

2) Decide between PWH (text) and PLWH (What is already known box)

Reply: We have corrected for consistency with PWH in the box.

3) The US measure of mg/dL is used throughout; simultaneous mmol/L equivalents for 3. 3. The US measure of mg/dL is used throughout; simultaneous mmol/L equivalents for UK-based BMJ Medicine??

Reply: We have converted the measures to mmol/L across the document (Page 3, Line 60; Page 4, Line 3; Page 4, Line 13-14; Table 1, Table 2)

4) The authors agree that the possibility of residual confounding is a limitation of the study. But it is not acknowledged in the discussion; it would seem wise to do so given the nature of the cohort, its time span, and the therapeutic evolution/s, both CV and HIV, that have occurred over the period.

Reply: It was mentioned in the methods and results, but we have now added this to the discussion (Page 10, Lines 41-42): "In addition, although we controlled for relevant factors or their proxies, unmeasured confounding variables may still exist, including diet, exercise, and excessive alcohol use."

This follows the paragraph discussing the lack of dose and adherence indicators, which are also part of the residual confounding factors

Reviewer #4: Dr. Neil Davies, University of Bristol

1) Pre-registration and analytic code - it would substantially strengthen the paper, and be useful for your readers to publish your protocol and code used to conduct this study. This is increasingly seen as a key part of reporting a study like this. You've done a great job on this analysis, and publishing the protocol and code would demonstrate this. However, I leave it to your and the editors' discretion.

Reply: Thank you for the feedback. We apology if this was not sufficiently addressed. Our study was conducted using data requested through the NA-ACCORD Concept Sheet process (https://naaccord.org/concept-sheet), which underwent internal review by the NA-ACCORD Working Group (WG) and Steering Committee (SC). These groups also had to review the manuscript prior to submission. These are part of the NAACCORD internal procedures (please see the procedure flow). Apart from this procedure, there were no additional pre-registrations. We have included the target trial protocol in Table 1 of the manuscript.

For the codes, we employed well-established causal estimation methods, and details on benefit-harm modeling are provided in Supplementary Materials (Page 9) and the codes are simply the models. In fact, 90% of the codes are about data processing, which vary based on data structure and are not meaningful without access to the dataset. However, we are happy to share the code with those interested in similar projects.
